# Effects of diverse resistance training modalities on performance measures in athletes: a network meta-analysis

**DOI:** 10.3389/fphys.2024.1302610

**Published:** 2024-02-01

**Authors:** Zhipeng Zhu, Haowen Wu, Longpeng Li, Mingyuan Jia, Dong Li

**Affiliations:** ^1^ College of Physical Education, Shanxi University, Taiyuan, Shanxi, China; ^2^ Department of Physical Education, Dong-A University, Busan, Republic of Korea; ^3^ Department of International Culture Education, Chodang University, Muan, Republic of Korea

**Keywords:** resistance training, complex training, lower limbs power, plyometric exercises, network meta-analysis

## Abstract

**Background:** Jumping ability is one of the necessary qualities for athletes. Previous studies have shown that plyometric training and complex training including plyometrics can improve athletes’ jumping ability. With the emergence of various types of complex training, there is uncertainty about which training method has the best effect. This study conducted a meta-analysis of randomized controlled trials of plyometric-related training on athletes’ jumping ability, to provide some reference for coaches to design training plans.

**Methods:** We systematically searched 3 databases (PubMed, Web of Science, and Scopus) up to July 2023 to identify randomized controlled trials investigating plyometrics related training in athletes. The two researchers conducted literature screening, extraction and quality assessment independently. We performed a network meta-analysis using Stata 16.

**Results:** We analyzed 83 studies and found that complex training, which includes high-intensity intervals and plyometric exercises, was the most effective method for improving squat jumps (SURCA = 96%). In the case of countermovement jumps a combination of electrostimulation and plyometric training yielded the best results (SURCA = 97.6%). Weightlifting training proved to be the most effective for the standing long jump (SURCA = 81.4%), while strength training was found to be the most effective for the five bounces test (SURCA = 87.3%).

**Conclusion:** Our current study shows that complex training performs more efficient overall in plyometric-related training. However, there are different individual differences in the effects of different training on different indicators (e.g., CMJ, SJ, SLJ, 5BT) of athletes. Therefore, in order to ensure that the most appropriate training is selected, it is crucial to accurately assess the physical condition of each athlete before implementation.

**Clinical Trial Registration:** https://www.crd.york.ac.uk/PROSPERO/, Registration and protocol CRD42023456402.

## 1 Introduction

Plyometric training is an effective method for improving physical fitness (Spurrs et al., 2003; Chelly et al., 2015; Sammoud et al., 2021). It not only enhances the specific performance of athletes but also improves overall health, learning of movement skills and injury prevention (Ramirez-Campillo et al., 2023). In athlete training, plyometric training is commonly used to exercise lower body strength, especially jumping ability, as jumping ability is crucial in many sports (Sheppard et al., 2008). The direction of jumping is related to specific sports (Sattler et al., 2012). For example, vertical jumping ability is applied in basketball for dunking and rebounding, while horizontal jumping ability reflects the performance of long jump athletes.

Typical plyometric exercises include vertical jumps, standing broad jumps, multiple jumps, single-leg jumps, rebound jumps and drop jumps (DJ) (Makaruk et al., 2020). The principle behind plyometric training improving jumping performance lies in its effective utilization of the stretch-shortening cycle (SSC) to enhance muscle strength and contraction velocity (Strate et al., 2021), achieving an optimal balance between strength and speed to generate maximum muscle output power. SSC can be divided into slow SSC (e.g., countermovement jump) and fast SSC (e.g., DJ) based on muscle contraction velocity and range of motion, which are applied in different competitive situations (Flanagan and Comyns, 2008).

As athletes’ abilities develop and they gradually adapt to training methods, individual plyometric training alone is no longer sufficient to meet their training needs. By combining plyometric training with other methods such as traditional resistance training or balance training, complex training theoretically allows for greater benefits (e.g., enhanced maximal strength, body control ability). However, many studies have shown that complex training is more time-efficient and effective than plyometric training (Arabatzi et al., 2010; Benito-Martínez et al., 2011; Zghal et al., 2019). Unfortunately, previous studies have compared different concepts of plyometric training (Strate et al., 2021), such as the use of elastic bands or increased resistance, to determine which is more effective in improving jumping ability, but there is no research comparing the effects of plyometric training with a variety of different complex training that includes plyometric exercises on jumping ability improvement.

While plyometric training has been proven to be an effective method for improving athletes’ jumping ability in various sports (Markovic and Newton, 2007; Bedoya et al., 2015; Arntz et al., 2022; Ramirez-Campillo et al., 2022), coaches and athletes also seek more efficient and safer training methods. Therefore, in our study, we aim to further verify and select the most optimal or better training methods through multiple evidence of network meta-analysis (NMA), providing a reference for coaches and athletes in their training.

Countermovement jump (CMJ), squat jump (SJ), standing long jump (SLJ), and 5-bounds test (5BT) are commonly used tests to evaluate an individual’s vertical and horizontal jumping ability and strength level (Michailidis et al., 2013; Michailidis et al., 2019; Negra et al., 2020). Therefore, this study will compare the effectiveness of plyometric training and complex training in improving jumping ability using the aforementioned indicators. Additionally, in this study, athletes refer to individuals who undergo systematic training, participate in professional competitive sports, and have the potential to become professional athletes in the future, including both youth and adult athletes, regardless of their skill level. The reasons for improving jump performance through plyometric training are clarified: (1) determining the effectiveness of different training methods associated with plyometric training and which one is more effective, and (2) determining if the quantity of plyometric exercises performed has an impact.

## 2 Materials and methods

### 2.1 Protocol and registration

The meta-analysis was conducted using the Cochrane Interventions Handbook for systematic reviews (Higgins et al., 2019). The study results will be reported in accordance with the Preferred Reporting Items for Systematic Reviews and Meta-Analyses (PRISMA) statement (Page et al., 2021). This network meta-analysis has been prospectively registered in PROSPERO under the registration number CRD42023456402.

### 2.2 Data sources and search strategy

Three databases (PubMed, Scopus, Web of Science) were searched for this study, with a search period from the inception of these databases until July 2023. The PICOS tool was used as the basis for the search strategy: (P) Population: healthy athletes; (I) Intervention: Plyometric training or compound training including Plyometric training; (C) Comparator: control group receiving regular training activities or resistance exercises; (O) Outcome: countermovement jump, squat jump, standing long jump, 5 bounces test; (S) Study type: randomized controlled trials. The search strategy can be found in Supplementary Appendix A.

### 2.3 Study selection

The literature was screened and excluded using the reference management tool Note Express. Firstly, two researchers independently searched for duplicates, non-randomized controlled trials, review articles, and other non-relevant studies based on the article titles. Then, the abstracts of the remaining articles were evaluated to determine which ones should be included in the study and which ones should be excluded. A full examination of the remaining papers was conducted before selecting further studies for inclusion. Throughout this process, all literature was reviewed individually and then compared to see if they were the same. Any discrepancies were resolved through group discussions.

### 2.4 Inclusion and exclusion criteria

The following criteria were used for inclusion in the literature review:

1) Participants: Athletes without injuries or diseases.
2) Study design: Randomized controlled trials with control groups receiving either regular training, resistance training, or plyometric training.
3) Clear reporting of outcome measures, including CMJ, SJ, SLJ, or 5BT.

The following criteria were used for exclusion:

1) Participants with injuries or diseases.
2) Data not reported or incomplete in the study.
3) Non-randomized controlled trials.
4) Duplicate articles.
5) Conference papers, review articles, and literature unrelated to the content of this study.

### 2.5 Data extraction

The extracted data were organized in a standardized Excel spreadsheet. Data from the included trials were independently extracted by two authors (DL and ZZ), and any discrepancies were resolved through group discussions. The following information was extracted from each study: author, year, participant characteristics, intervention features, and relevant indicators. Common indicators reflecting lower limb jumping ability were selected as outcome measures. Horizontal jumping ability included SLJ and 5BT, while vertical jumping ability included CMJ and SJ. If a study included multiple related or similar indicators, a representative indicator was prioritized (e.g., choosing CMJ over CMJ with arms to reduce the influence of arm swing). When encountering ambiguous intervention results presented graphically, Engauge Digitizer software was used to extract the data. In cases where standard deviation was not provided, we calculated it using a 95% confidence interval. In this study, strength training is categorized based on its movement form into weightlifting training (WL), traditional resistance training (ST), and plyometric training (PT). Furthermore, to differentiate between training methods, we consider PT to consist of two or more jumping exercises (e.g., hurdle jump, hop. jumps). If only one type of jumping movement is involved, we directly use its abbreviation (e.g., drop jump, DJ). If plyometric training is combined with non-plyometric training, we will use the abbreviations of each component to name it (e.g., weightlifting training combined with plyometric training, WL + PT), and categorize it as complex training, regardless of the quantity of plyometric exercises or other components outside of plyometric training.

### 2.6 Quality assessment

The risk of bias was assessed using the Cochrane Risk of Bias assessment tool in Review Manager 5.4 software. This tool evaluates the quality of studies based on seven domains: 1. Random sequence generation; 2. Allocation concealment; 3. Blinding of participants and personnel; 4. Blinding of outcome assessment; 5. Incomplete outcome data; 6. Selective reporting; and 7. Other biases.

### 2.7 Statistical analysis

This study will report continuous variables as standardized mean differences (SMD) due to variations in measurement methods and techniques across different studies, along with their corresponding 95% confidence intervals (CI) and analyses. We will use a random-effects model for the analysis rather than a fixed-effects model, as there is expected heterogeneity among the studies (Jackson et al., 2011).

Following the PRISMA-NMA guidelines, we will utilize Stata software (version 16) within a Bayesian framework for Markov chain Monte Carlo simulation, chain aggregation, and analysis of NMA data (Moher et al., 2015; Vats et al., 2019). The node-splitting method will be utilized in Stata software to quantify and assess the consistency between indirect comparisons and direct comparisons. If the *p*-value is greater than 0.05, it is considered that the consistency test has passed (Salanti et al., 2011; Li et al., 2023a). Stata software will also be used for generating and describing the network plot of various interventions. The Surface Under the Cumulative Ranking curve (SUCRA) values will be used to determine the relative rankings of interventions. SUCRA values range from 0 to 100, with values closer to 0 indicating poorer effectiveness and values closer to 100 indicating greater effectiveness. While SUCRA represents the effectiveness of interventions in an acceptable percentage, caution should also be exercised when interpreting it, unless there are truly significant differences between different trials. At the same time, SUCRA only takes into account the relative effect between different interventions and does not provide information about the absolute effect size of each intervention. Therefore, when interpreting SUCRA results, it is recommended to consider other information such as the original effect size, confidence intervals, *etc.*, in order to obtain a more comprehensive assessment. League tables can serve as a supplement to assess the effects between different interventions using effect sizes and confidence intervals. Funnel plots can provide an intuitive assessment of bias in small-study effects due to their symmetry (Li et al., 2023b), although this may introduce publication bias in NMA. The potential impact of publication bias on study outcomes will also be investigated using Egger’s and Begg’s tests, and if publication bias is found, it will be explored through sensitivity analyses to identify potential sources of bias.

## 3 Results

### 3.1 Study and identification and selection

**FIGURE 1:**
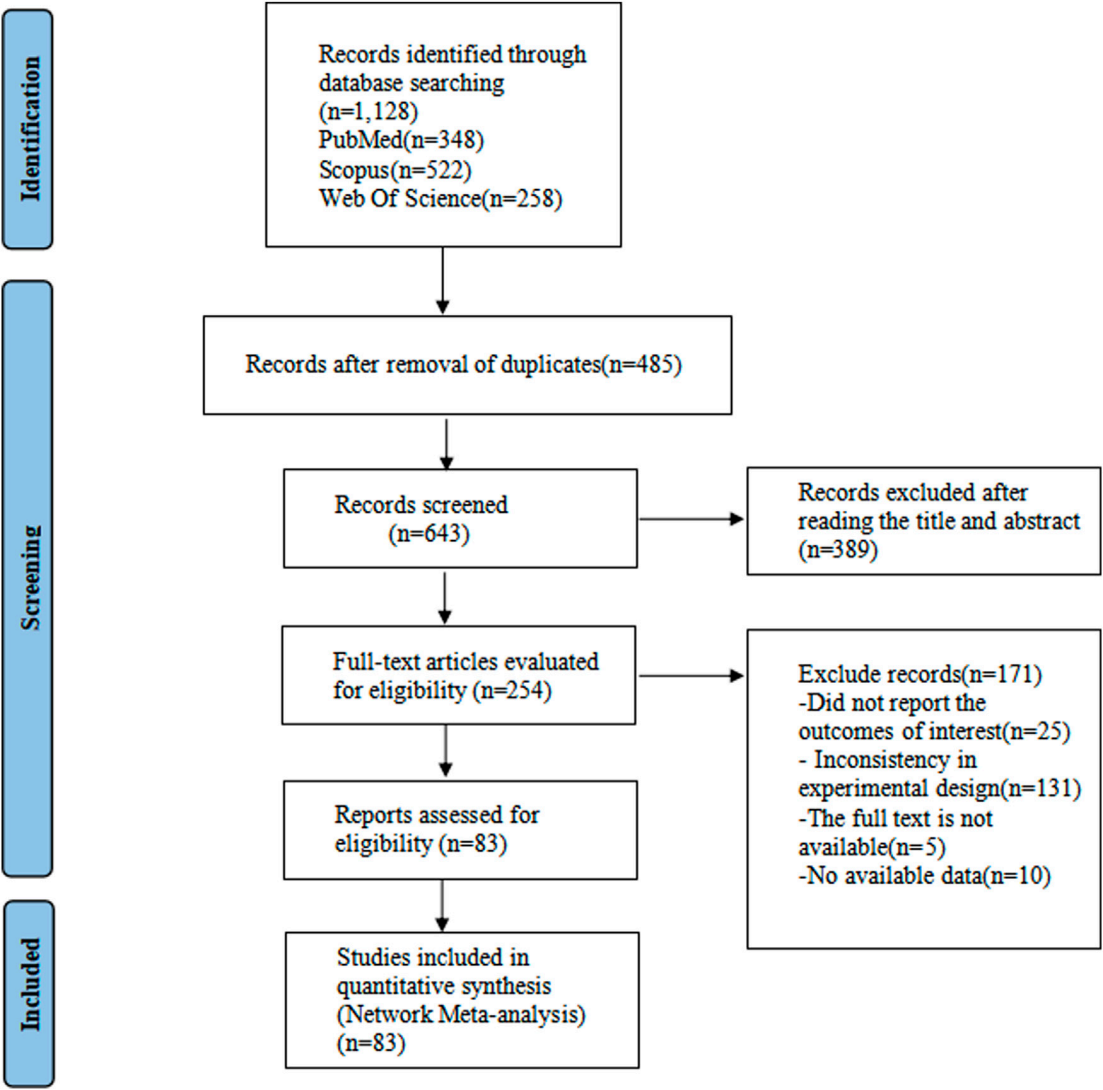
PRISMA flow diagram of the study process.

The study initially retrieved a total of 1,128 articles from three databases. After removing duplicate articles, there were 485 articles remaining. Based on the titles and abstracts, 389 articles were excluded. After reading the full texts of the remaining 254 articles, an additional 171 articles were excluded (due to unavailability of full text, lack of relevant data, or inconsistency with the study’s content). Ultimately, 83 articles were included in the study (Figure 1) (Matavulj et al., 2001; Spurrs et al., 2003; Turner et al., 2003; Toumi et al., 2004; Martel et al., 2005; Ronnestad et al., 2008; Santos and Janeira, 2008; Carlson et al., 2009; Thomas et al., 2009; Berryman et al., 2010; Khlifa et al., 2010; Benito-Martínez et al., 2011; Voelzke et al., 2012; Michailidis et al., 2013; Pienaa and Coetzee, 2013; Chelly et al., 2014; Garcia-Pinillos et al., 2014; Ozbar et al., 2014; Ramírez-Campillo et al., 2014; Redondo et al., 2014; Zribi et al., 2014; Attene et al., 2015; Chelly et al., 2015; Sáez De Villarreal et al., 2015; Hall et al., 2016; Ramirez-Campillo et al., 2016; Rodríguez-Rosell et al., 2016; Agostini et al., 2017; Chtara et al., 2017; Dæhlin et al., 2017; Neves Da Silva et al., 2017; Rodríguez-Rosell et al., 2017; Rosas et al., 2017; Asadi et al., 2018; Beato et al., 2018; Chatzinikolaou et al., 2018; Fischetti et al., 2018; Hernandez et al., 2018; Idrizovic et al., 2018; Ita and Guntoro, 2018; Latorre Román et al., 2018; Makhlouf et al., 2018; Ramirez-Campillo et al., 2018; Hammami et al., 2019a; Hammami et al., 2019b; Jlid et al., 2019; Meszler and Váczi, 2019; Michailidis et al., 2019; Ramirez-Campillo et al., 2019; Sammoud et al., 2019; Zghal et al., 2019; Bouteraa et al., 2020; Drouzas et al., 2020; Hammami et al., 2020a; Hammami et al., 2020b; Jlid et al., 2020; Ramirez-Campillo et al., 2020a; Ramirez-Campillo et al., 2020b; Vera-Assaoka et al., 2020; Aloui et al., 2021; Chaabene et al., 2021; Freitas et al., 2021; Hammami et al., 2021; Maciejczyk et al., 2021; Padrón-Cabo et al., 2021; Palma-Muñoz et al., 2021; Porrati-Paladino and Cuesta-Barriuso, 2021; Sammoud et al., 2021; Sáez De Villarreal et al., 2021; Falch et al., 2022; Kaabi et al., 2022; Lum et al., 2022; Nonnato et al., 2022; Panda et al., 2022; Rojano Ortega et al., 2022; Sanchez et al., 2022; Villalba et al., 2022; Aztarain-Cardiel et al., 2023; Brini et al., 2023; Cabrejas et al., 2023; Lum et al., 2023; Marzouki et al., 2023).

### 3.2 Characteristics of the included studies

This study includes 83 randomized controlled trials, with a total of 2,597 participants. All studies were published between 2001 and July 2023. The majority of participants were soccer players, with 39 studies focusing on this population. The age of the athletes ranged from 8 to 43, although the majority of studies concentrated on the age range of 10–30. The male proportion was higher among the participants in the studies.

Generally, intervention strategies can be categorized into two main types: Plyometric training and non-plyometric training (WL; ST; conventional training, ct). Plyometric training can be further classified into three subcategories: complex training, PT, and plyometric training with single exercise. The number of jumps per training session ranged from 35 to 260, and the intervention frequency varied from 1 to 4 times per week. The intervention period ranged from 4 to 12 weeks. Additionally, in almost all studies, participants were instructed to exert maximum effort during each jump. Vertical jumping ability was predominantly measured using CMJ, while horizontal jumping ability was often measured using the SLJ. For more details, please refer to Supplementary Appendix B. There are many different distinctions in the literature about elite or subelite athletes. In this paper, we consider the training years of athletes as an indicator to reflect the athletic level of athletes to some extent.

### 3.3 Quality assessment of the included studies

**FIGURE 2:**
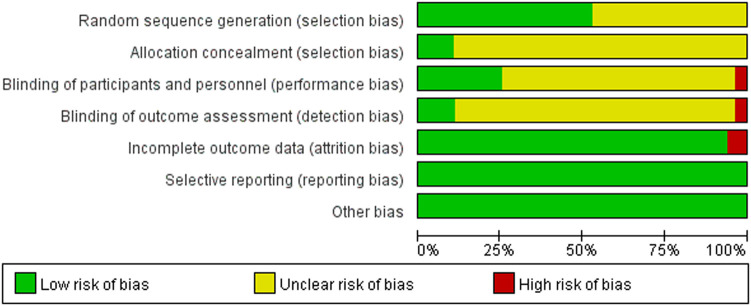
Methodological quality of included studies.

44 studies concluded that the risk of random sequence generation is low, while 39 studies did not clearly disclose how the random sequence was generated, leading to uncertainty in risk assessment. Among the 83 articles, 9 were considered to have low risk of allocation concealment bias, while 74 did not clearly state their allocation concealment methods, hence carrying some risk. Due to the difficulties in implementing interventions under double-blind conditions, only 21 studies believed that the bias risk was low for both researchers and participants, while 3 studies were associated with higher bias risk. Therefore, the overall bias risk of this standard is relatively high. Regarding the assessment of blinding, 9 studies employed professional or blinded assessors, 3 studies did not, and 71 studies did not specify the method of result evaluation, indicating higher risk in the evaluation method. Out of the 78 studies, the number of participants after intervention remained consistent or nearly consistent with the baseline, and complete result data were reported. In contrast, 5 studies showed some differences in participant numbers after intervention compared to the baseline, indicating lower risk. No evidence of selective reporting bias or other forms of bias risk were found in any of the studies. Detailed information on the assessment of bias in the included literature can be found in Figure 2.

### 3.4 Network meta-analysis

**FIGURE 3:**
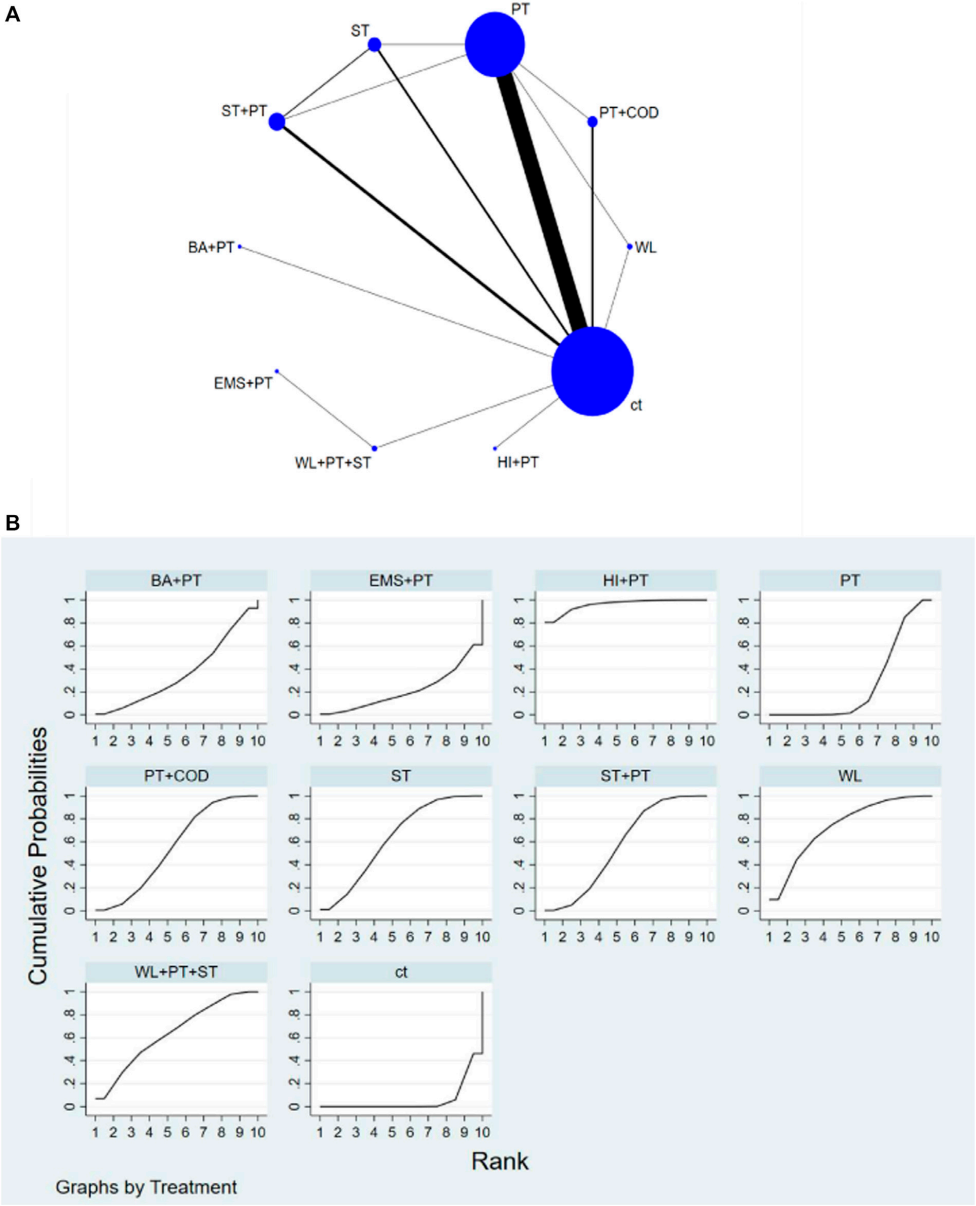
**(A)** NMA figure for SJ. **(B)** SUCRA plot for SJ.

**FIGURE 4:**
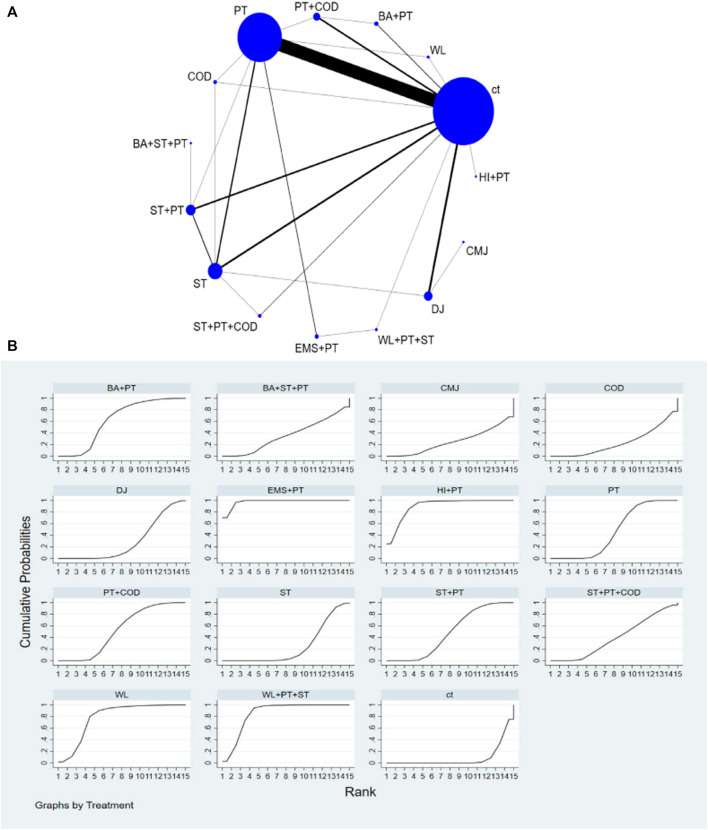
**(A)** NMA figure for CMJ. **(B)** SUCRA plot for CMJ.

**FIGURE 5:**
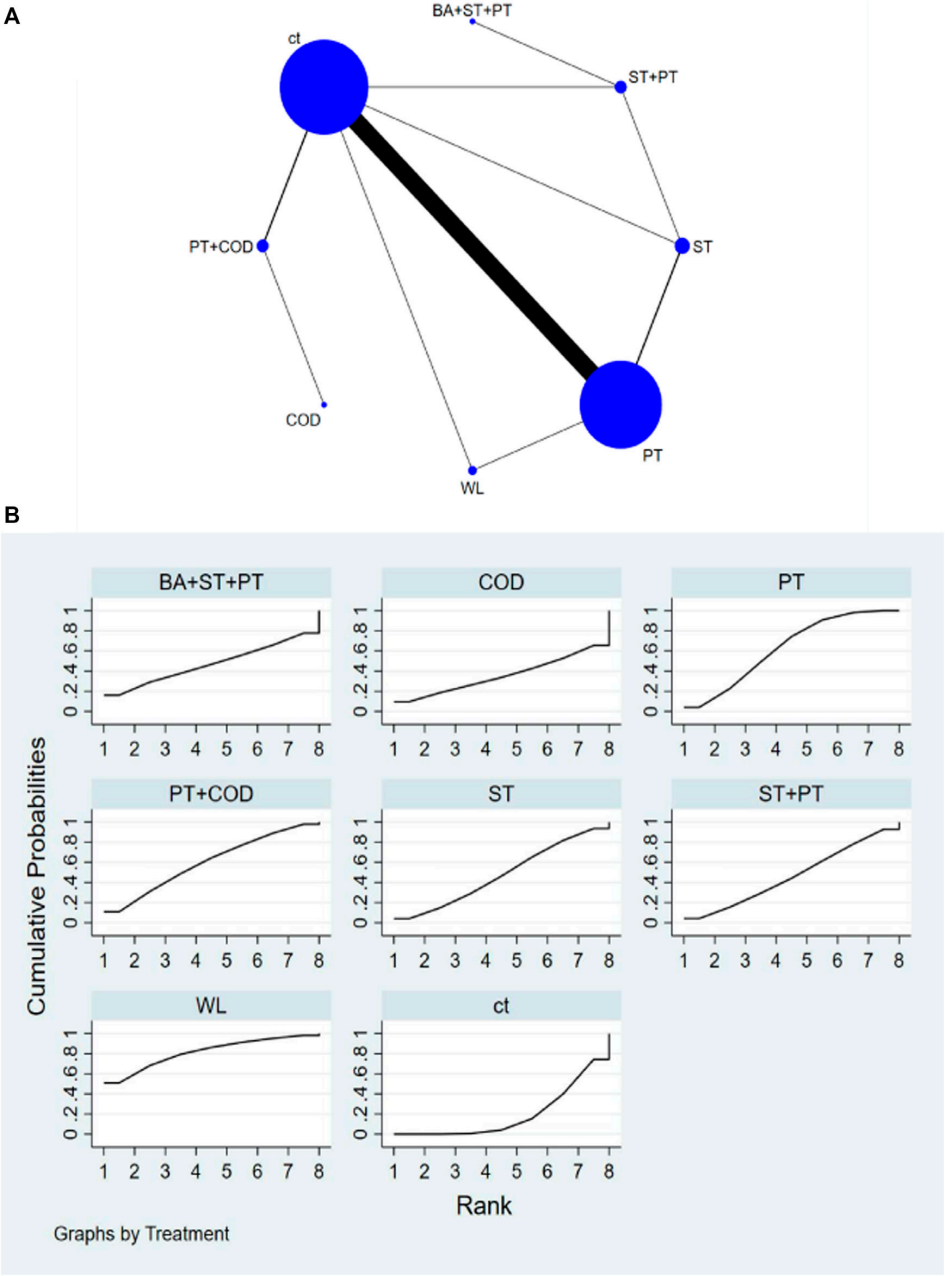
**(A)** NMA figure for SLJ. **(B)** SUCRA plot for SLJ.

**FIGURE 6:**
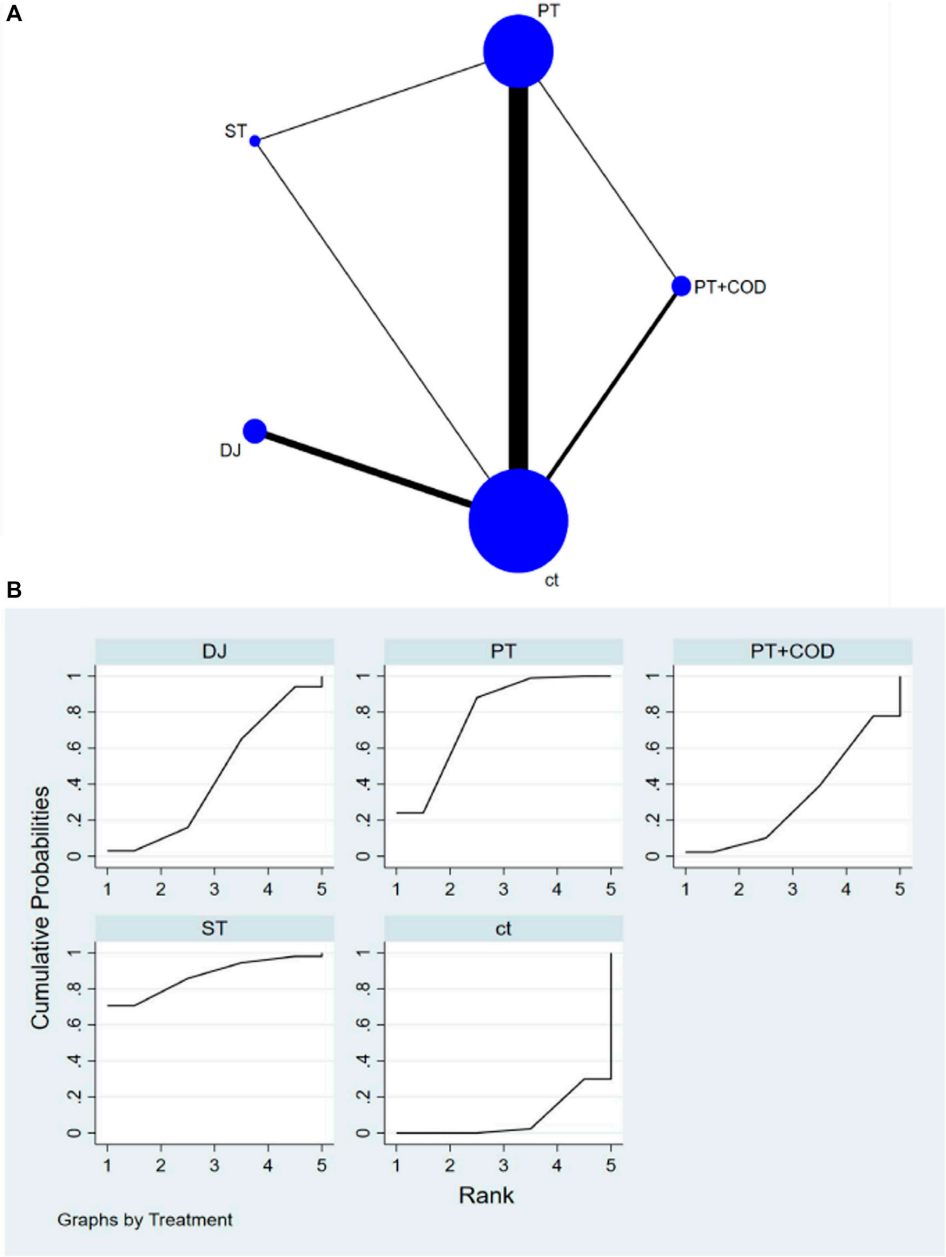
**(A)** NMA figure for 5BT. **(B)** SUCRA plot for 5BT.

The network diagram illustrating various training interventions can be found in Figures 3–6 A. In the diagram, circles represent different intervention measures, the size of the circles corresponds to the number of participants, and the thickness of the lines represents the number of studies.

#### 3.4.1 Improvement in SJ among athletes

Based on the results of NMA, the following interventions were found to significantly improve SJ: High-intensity interval training combined with plyometrics (HI + PT) [SMD = 2.75, 95% CI=(1.36,4.14)], WL [SMD = 1.7, 95% CI=(0.61,2.78)], Complex training consists of weightlifting, traditional resistance, and plyometrics (WL + PT + ST) [SMD = 1.46, 95% CI=(0.10,2.83)], ST [SMD = 1.42, 95% CI=(0.73,2.10)], traditional resistance training combined with plyometrics (ST + PT) [SMD = 1.29, 95% CI=(0.74,1.83)], Plyometric training combined with change of direction training (PT + COD) [SMD = 1.25, 95% CI=(0.65,1.86)], Balance training combined with plyometric training (BA + PT) [SMD = 0.81, 95% CI=(-0.46,2.07)], PT [SMD = 0.73, 95% CI=(0.50,0.97)], Electrostimulation combined with plyometric training (EMS + PT) [SMD = 0.24, 95% CI=(-1.76,2.24)]. Compared to conventional training (ct), all types of interventions showed better improvement in SJ. Based on SUCRA analysis (Figure 3B; Table 1), the probability ranking of various interventions in improving SJ indicates a higher likelihood for HI + PT (SUCRA = 96%). Pairwise comparisons between different training interventions will be shown in Supplementary Appendix C1.

**TABLE 1:** Summary of interventions SURCA and rankings.

Intervention	SJ		CMJ		SLJ		5BT	
	SURCA	Rank	SURCA	Rank	SURCA	Rank	SURCA	Rank
EMS+PT	21.3	9	97.6	1				
HI+PT	96	1	90.3	2				
WL+PT+ST	64	3	85.5	3				
WL	73.5	2	78.8	4	81.4	1		
BA+PT	36.3	7	62.2	5				
PT+COD	55.5	6	52.8	6	59.9	3	32.4	4
ST+PT	57.3	5	48.6	7	46.5	6		
PT	27.1	8	47	8	62.7	2	77.8	2
ST+PT+COD			40	9				
BA+ST+PT			35.7	10	47	5		
DJ			29.5	11			44.5	3
CMJ			25.4	12				
ST	63.1	4	24.9	13	47.8	4	87.3	1
COD			23.3	14	35.5	7		
ct	5.8	10	8.5	15	19.2	8	8.1	5

#### 3.4.2 Improvement in CMJ among athletes

Based on the results of NMA, the following interventions were found to significantly improve CMJ: EMS + PT [SMD = 3.45, 95% CI=(2.52,4.38)], HI + PT [SMD = 2.86, 95% CI=(1.32,4.41)], WL + PT + ST [SMD = 2.41, 95% CI=(1.23,3.60)], WL [SMD = 1.95, 95% CI=(0.72,3.18)], BA + PT [SMD = 1.13, 95% CI=(0.38,1.87)], PT + COD [SMD = 0.88, 95% CI=(0.37,1.39)], ST + PT [SMD = 0.78, 95% CI=(0.27,1.29)], PT [SMD = 0.75, 95% CI=(0.55,0.94)], Complex training consists of traditional resistance, plyometrics, and change of direction (ST + PT + COD) [SMD = 0.64, 95% CI=(-0.29,1.58)], Complex training consists of balance, traditional resistance and plyometrics (BA + ST + PT) [SMD = 0.49, 95% CI=(-1.01,2.00)], Drop Jump (DJ) [SMD = 0.45, 95% CI=(-0.02,0.91)], CMJ [SMD = 0.16, 95% CI=(-1.53,1.86)], ST [SMD = 0.37, 95% CI=(-0.04,0.78)], Change of direction (COD) [SMD = 0.23, 95% CI=(-0.96,1.43)]. Compared to the CT, all types of interventions showed better improvement in CMJ. According to SUCRA analysis (Figure 4B; Table 1), the probability ranking of various interventions in improving CMJ suggests a higher likelihood for EMS + PT (SUCRA = 97.6%). Pairwise comparisons between different training interventions will be shown in Supplementary Appendix C2.

#### 3.4.3 Improvement in SLJ among athletes

Based on the results of NMA, the following interventions were found to significantly improve SLJ: WL [SMD = 1.24, 95% CI=(-0.13,2.61)], PT [SMD = 0.7, 95% CI=(0.33,1.06)], PT + COD [SMD = 0.69, 95% CI=(-0.42,1.79)], ST [SMD = 0.46, 95% CI=(-0.52,1.44)], BA + ST + PT [SMD = 0.43, 95% CI=(-1.56,2.42)], ST + PT [SMD = 0.43, 95% CI=(-0.76,1.62)], COD [SMD = 0.14, 95% CI=(-1.85,2.14)]. Compared to the CT, all types of interventions showed better improvement in SLJ. According to SUCRA analysis (Figure 5B; Table 1), the probability ranking of various interventions in improving SLJ suggests a higher likelihood for WL (SUCRA = 81.4%). Pairwise comparisons between different training interventions will be shown in Supplementary Appendix C3.

#### 3.4.4 Improvement in 5BT among athletes

Based on the results of NMA, the following interventions were found to significantly improve 5BT: ST [SMD = 1.39, 95% CI=(0.06,2.71)], PT [SMD = 0.98, 95% CI=(0.56,1.40)], DJ [SMD = 0.49, 95% CI=(-0.14,1.12)], PT + COD [SMD = 0.31, 95% CI=(-0.51,1.12)]. Compared to the CT, all types of interventions showed better improvement in 5BT. According to SUCRA analysis (Figure 6B; Table 1), the probability ranking of various interventions in improving 5BT suggests a higher likelihood for ST (SUCRA = 87.3%). Pairwise comparisons between different training interventions will be shown in Supplementary Appendix C4.

### 3.5 Publication bias test

**FIGURE 7:**
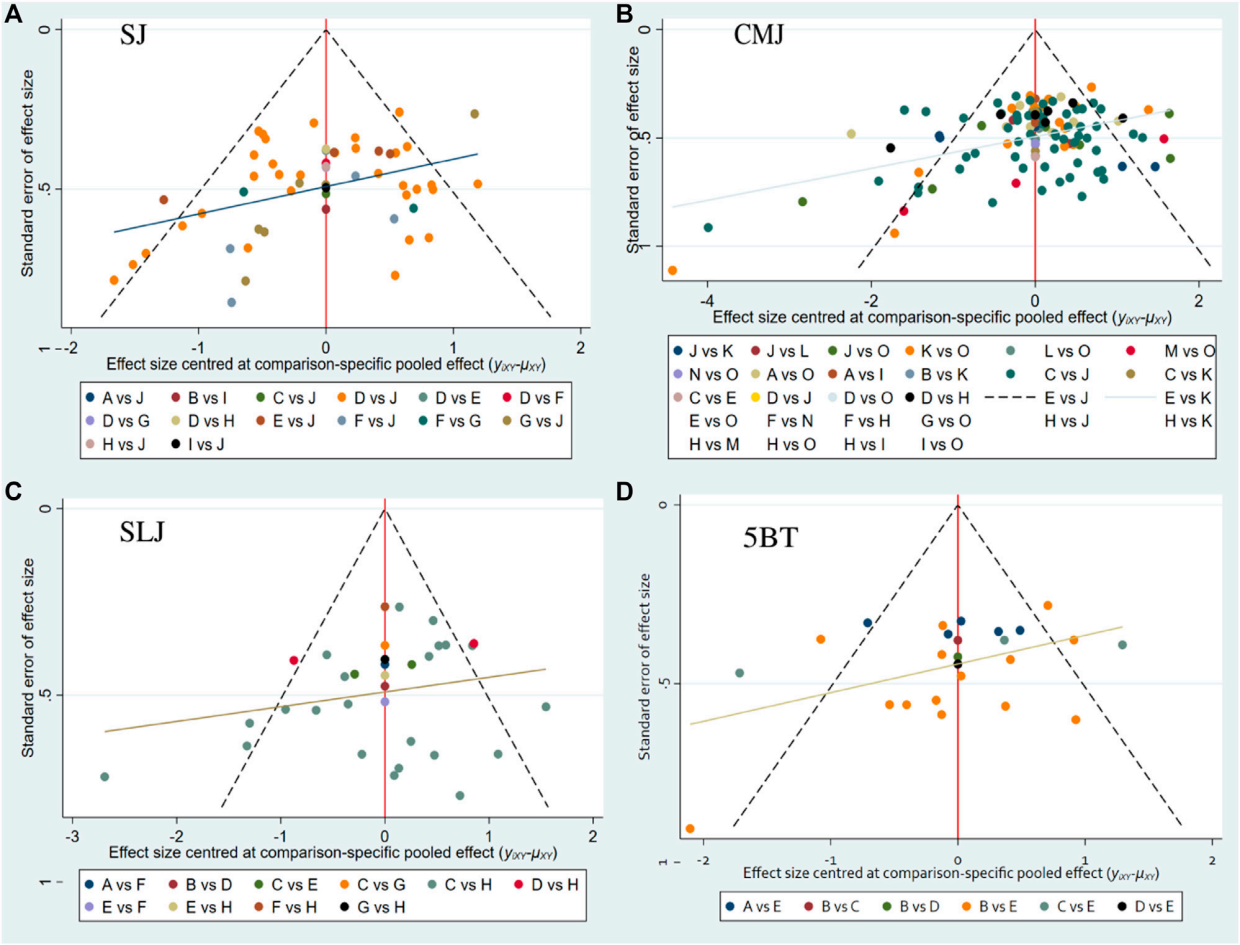
Funnel plot.

As shown in Figure 7, funnel plots were used to assess publication bias. Visual inspection of the funnel plots for all outcomes suggested the presence of publication bias, which was confirmed by Begg’s and Egger’s tests. Sensitivity analysis can be found in Supplementary Appendix D.

## 4 Discussion

### 4.1 Findings

In this study, we conducted a comparative analysis of 15 training interventions to improve jump performance by combining literature search. We found 83 studies that met the inclusion criteria. Consistent with previous research, our analysis focused on jump performance indicators including vertical jumps (SJ and CMJ) and horizontal jumps (SLJ and 5BT). The sample sizes for SJ, CMJ, SLJ, and 5BT were 1,198, 2,415, 728, and 620 athletes, respectively. As shown in the network relationship graph, the majority of studies and participants were centered around PT and ST, with fewer studies examining other interventions.

The reason for including a large number of PT and ST studies in our analysis is twofold. Firstly, PT and ST are the most commonly used methods in athletes’ daily training. Secondly, these methods involve various variations such as randomized training exercises order, progressive loading, vertical or horizontal movements, and concentric or eccentric muscle actions. Previous research has shown that these variations can improve training outcomes compared to conventional plyometric training. Although we categorized them as PT in this study (as they did not involve changes in the movement composition), publication bias is acceptable. Additionally, we assumed that there is a limit to the effectiveness of each training method and included as many studies as possible to define a general range. If the rankings were similar to other interventions, we considered that certain variables could be adjusted to achieve similar or even better results. Recent research (Ramirez-Campillo et al., 2023) indicates that maturity does not affect the effectiveness of plyometric training. Given that most of the athletes in our study were in the pre-and post-pubertal period, we believe that the influence of age deviation on the study is minimal.

For studies using SJ as the outcome measure, we compared 10 interventions and found that HI + PT was the most effective method. However, the effectiveness of high-intensity interval training alone in improving jump performance is still controversial and may be influenced by the training status of the participants (Hammami et al., 2021). The improvement in jump performance for elite athletes is mainly attributed to the plyometric training component (Hammami et al., 2021). As a measure of lower limb strength, it is incredible that HI + PT ranked first. The second and third rankings were WL and WL + PT + ST, respectively. Consistent with Soufiane’s research, WL has been shown to be superior to ST in improving both strength and jump performance (Kaabi et al., 2022). The superior effects of WL + PT can be explained by their complementary benefits. Additionally, ST has been shown to be more effective in improving maximal strength compared to PT alone (Negra et al., 2020). PT + ST was superior to PT in improving SJ (Zghal et al., 2019). We speculate that WL and ST are effective methods for improving SJ, and that plyometric training should be combined with weightlifting for even more effective improvements. Further research is needed to validate these findings.

For studies using CMJ as the outcome measure, we compared 15 interventions and found that EMS + PT was the most effective method. While there is evidence that electromyostimulation (EMS) alone can improve vertical jump height (Benito-Martínez et al., 2011), the improvement in CMJ performance is primarily attributed to the improvement in stretch-shortening cycle (SSC). EMS + PT is effective because it leverages the elasticity and neural adaptation of the SSC and enhances muscle activation through electromyographic enhancement (Voelzke et al., 2012). Furthermore, the order of combining EMS and PT can influence the improvements in CMJ performance. When EMS is performed before PT, the over-stimulated muscles are more activated and have better receptive capacity, allowing for higher training loads in subsequent plyometric training, resulting in improved training quality and benefits, with higher time efficiency. The rankings for CMJ were similar to SJ, with HI + PT, WL + PT + ST, and WL ranked second, third, and fourth, respectively. The difference is that the rankings for WL + PT + ST were higher than WL, which is consistent with the impact of CMJ performance mentioned earlier. The adaptation mechanisms for WL + PT + ST and EMS + PT may differ. WL + PT + ST emphasizes concentric force of the extensor muscles, while EMS + PT improves the rate of force development (RFD) and positive adaptation of the SSC (Voelzke et al., 2012). In fact, PT can effectively improve the utilization of the SSC, and the combination of WL and ST can balance speed and strength, resulting in excellent power performance (Chatzinikolaou et al., 2018). Additionally, EMS training has been shown to result in superior strength improvements compared to training involving active muscle contractions (Benito-Martínez et al., 2011). It is reasonable to infer that EMS + PT could potentially be superior to WL + PT + ST, which is consistent with our study results.

For studies using SLJ as the outcome measure, we compared eight interventions and found that WL was the most effective training method. WL movements involve maximal speed, which may lead to greater motor-unit synchronization (Kaabi et al., 2022). Although WL may not improve strength qualities as much as ST, it exhibits higher speed-strength performance (Chatzinikolaou et al., 2018). Therefore, the positive effects of WL on jump performance, both in the vertical and horizontal directions, are undeniable and consistent with our study results. PT ranked second, which is consistent with previous studies that showed its superior effects compared to ST (Negra et al., 2020; Falch et al., 2022).

For studies using 5BT as the outcome measure, we compared five interventions and found that ST was the most effective training method. However, in Yassine’s study (Negra et al., 2020), ST alone did not show specific improvements in jump performance. Improvements in jump performance could be achieved effectively and rapidly by primarily focusing on increasing maximal strength, which may be related to the participants’ individual conditions. Additionally, due to the limited number of studies included for ST, we find PT to be a more convincing method for improving 5BT.

Overall, compared to PT, complex training has similar or better effects on vertical jump performance and PT is more effective for horizontal jump performance. PT is superior to single-exercise plyometric training, which is consistent with previous research (Kaabi et al., 2022). In addition to plyometric training, WL, ST, and EMS have positive effects on jump performance, and complex training combining PT with these modalities has the best improvement effects. It is worth noting that some training interventions have significant individual characteristics and are influenced by the participants’ training level, even within the athlete population. However, the selection of these training methods has been defined by their specific conditions of use, such as the high technical demands of weightlifting movements, which are not suitable for novice athletes, and the potential early fatigue that can be caused by including ST in complex training for junior athletes. Importantly, although some complex training interventions have higher thresholds, combining PT with balance training or COD appears to not reduce the benefits of PT and lead to more functional improvements, such as sprint speed and body balance control. This is highly meaningful for practical training across various athlete levels. Currently, it seems that complex training interventions with more components may have a detrimental effect on jump performance improvement.

### 4.2 Strengths and limitations

Firstly, our analysis included 2,597 athletes from 83 studies, resulting in a relatively large sample size. We conducted a network meta-analysis to evaluate the effects of different plyometric related training on jump performance in athletes. Through direct and indirect comparative evidence analysis, we identified and included a total of 15 types of training divided into 2 categories, providing comprehensive recommendations for coaches on the application of plyometric training. Secondly, it is important to note that our findings are not without limitations. Despite our efforts to include as many relevant studies as possible and control for heterogeneity in the included studies by considering their original data, certain types of training have limited existing research, and unavoidable variations exist between studies (e.g., athletes’ training levels, whether plyometric training is incorporated into regular training, training facilities). Therefore, readers must interpret the results with caution. Lastly, the outcome measures SJ, CMJ, SLJ, and 5BT represent jump performance in a general sense. Improvements in jump performance are influenced by multiple factors, and future research could explore more specific biological indicators such as strength and speed at different muscle contraction phases.

Plyometric training stands out as an exceptionally effective and practical method in sports training, offering a pathway to enhance athletes’ strength and speed capabilities. Complex training, which integrates plyometric training with other training methods, presents an efficient approach to augmenting athletes’ training benefits and improving overall training efficiency. However, there remains a scarcity of high-quality literature on the performance-enhancing effects of complex training, specifically involving plyometric training. In the current era, where the demand for diversified and scientifically efficient training in sports is growing, complex training emerges as a future development trend. Future complex training should prioritize the design and arrangement of training content, emphasizing the strengthening of connections between different training methods.

## 5 Conclusion

Our current research suggests that complex training has the potential to be more effective in plyometric-related training. However, different training interventions have varying individual differences in their effects on athletes’ performance indicators. Therefore, accurately assessing each athlete’s training level prior to implementation is crucial to maximize training benefits and ensure the selection of the most appropriate training intervention.

## Data Availability

The original contributions presented in the study are included in the article/Supplementary Material, further inquiries can be directed to the corresponding author.
