# Supplementary material for: Effects of diverse resistance training modalities on performance measures in athletes: a network meta-analysis
Source: Front Physiol. 2024 Feb 1;15:1302610. doi: 10.3389/fphys.2024.1302610 (PMC10870238; doi:10.3389/fphys.2024.1302610)

**Supplementary Appendix A.** Search strategy

A.1. Search strategy on Web of Science

|  | Search strategy | Search result |
| --- | --- | --- |
| 1 | (((((((((((((((((((((((((((((TS=(Plyometric Exercise)) OR TS=(Plyometric)) OR TS=(Exercise, Plyometric)) OR TS=(Exercises, Plyometric)) OR TS=(Plyometric Exercises)) OR TS=(Plyometric Drill))) OR TS=(Drill, Plyometric)) OR TS=(Plyometric Drills)) OR TS=(Plyometric Training)) OR TS=(Plyometric Trainings)) OR TS=(Training, Plyometric)) OR TS=(Trainings, Plyometric)) OR TS=(Stretch-Shortening Exercise)) OR TS=(Exercise, Stretch-Shortening)) OR TS=(Exercises, Stretch-Shortening)) OR TS=(Stretch Shortening Exercise)) OR TS=(Stretch-Shortening Exercises)) OR TS=(Stretch-Shortening Cycle Exercise)) OR TS=(Cycle Exercise, Stretch-Shortening)) OR TS=(Cycle Exercises, Stretch-Shortening)) OR TS=(Exercise, Stretch-Shortening Cycle)) OR TS=(Exercises, Stretch-Shortening Cycle)) OR TS=(Stretch Shortening Cycle Exercise)) OR TS=(Stretch-Shortening Cycle Exercises)) OR TS=(Stretch-Shortening Drill)) OR TS=(Drill, Stretch-Shortening)) OR TS=(Drills, Stretch-Shortening)) OR TS=(Stretch Shortening Drill)) OR TS=(Stretch-Shortening Drills) | 2042 |
| 2 | ((TS=(randomized controlled trial)) OR TS=(randomized)) OR TS=(placebo) | 1108887 |
| 3 | #1 AND #2 | 258 |

A.2. Search strategy on Pubmed

((("Plyometric Exercise"[Mesh]) OR ((((((((((((((((((((((((((((Exercise, Plyometric[Title/Abstract]) OR (Exercises, Plyometric[Title/Abstract])) OR (Plyometric Exercises[Title/Abstract])) OR (Plyometric Drill[Title/Abstract])) OR (Drill, Plyometric[Title/Abstract])) OR (Drills, Plyometric[Title/Abstract])) OR (Plyometric Drills[Title/Abstract])) OR (Plyometric Training[Title/Abstract])) OR (Plyometric Trainings[Title/Abstract])) OR (Training, Plyometric[Title/Abstract])) OR (Trainings, Plyometric[Title/Abstract])) OR (Stretch-Shortening Exercise[Title/Abstract])) OR (Exercise, Stretch-Shortening[Title/Abstract])) OR (Exercises, Stretch-Shortening[Title/Abstract])) OR (Stretch Shortening Exercise[Title/Abstract])) OR (Stretch-Shortening Exercises[Title/Abstract])) OR (Stretch-Shortening Cycle Exercise[Title/Abstract])) OR (Cycle Exercise, Stretch-Shortening[Title/Abstract])) OR (Cycle Exercises, Stretch-Shortening[Title/Abstract])) OR (Exercise, Stretch-Shortening Cycle[Title/Abstract])) OR (Exercises, Stretch-Shortening Cycle[Title/Abstract])) OR (Stretch Shortening Cycle Exercise[Title/Abstract])) OR (Stretch-Shortening Cycle Exercises[Title/Abstract])) OR (Stretch-Shortening Drill[Title/Abstract])) OR (Drill, Stretch-Shortening[Title/Abstract])) OR (Drills, Stretch-Shortening[Title/Abstract])) OR (Stretch Shortening Drill[Title/Abstract])) OR (Stretch-Shortening Drills[Title/Abstract])))) AND (randomized controlled trial[Publication Type] OR randomized[Title/Abstract] OR placebo[Title/Abstract]) Filters: Randomized Controlled Trial

A.3. Search strategy on SCOPUS

( TITLE-ABS-KEY ( randomized AND controlled AND trial ) OR TITLE-ABS-KEY ( randomized ) OR TITLE-ABS-KEY ( placebo ) ) AND ( TITLE-ABS-KEY ( plyometric ) OR TITLE-ABS-KEY ( stretch-shortening ) )

**Supplementary Appendix B.** Summary table of included reviews.

B.1. Characteristics of participants and classification of interventions.

| Study | N (IG; CG) | Age | Gender | Sports | Period | Training experience（year） | Intervention (IG) | | | Intervention (CG) | | | Outcomes |
| --- | --- | --- | --- | --- | --- | --- | --- | --- | --- | --- | --- | --- | --- |
|  |  |  |  |  |  |  | Intervention content | Number of jumps/session,frequency/week,number of weeks | Type | Intervention content | Number of jumps/session,frequency/week,number of weeks | Type |  |
| Barbara et al., 2017 | 15；15 | 15.2±1.5 | female | rhythmic gymnastics | preparatory,competitive | 7.5±1.6 | jumps | NR,2-3,12 | PT | regular training | 12weeks | ct | CMJ, SLJ |
| Abbas et al., 2018 | 10；10 | 11.5±0.8 | male | soccer | pre-season | 2< | drop jump | 60,2,6 | DJ | regular training | 6weeks | ct | CMJ |
|  | 10；10 | 14.0±0.7 | male | soccer | pre-season | 2< | drop jump |  | DJ | regular training |  | ct |  |
|  | 10；10 | 16.6±0.6 | male | soccer | pre-season | 2< | drop jump |  | DJ | regular training |  | ct |  |
| Bent et al., 2008 | 6；15 | 22±2.5 | male | soccer | NR | NR | half squat, jumps | NR,2,7 | ST+PT | half squat | NA,2,7 | ST | SJ, CMJ |
|  |  |  |  |  |  |  |  |  |  | regular training | 7weeks | ct |  |
| Fabián et al., 2017 | 8；9 | 22.8±2.1 | female | soccer | NR | 7.5±3.1 | jumps | 140-260,2,6 | PT | regular training | 6weeks | ct | SJ, CMJ |
| Eduardo et al., 2021 | 10；30 | 13.57±1.39 | male | basketball | in-season | 4.85±1.86 | jumps | NR,2,7 | PT | full squat, Rise to bench, Calf muscle | NA,2,7 | ST | CMJ |
|  |  |  |  |  |  |  |  |  |  | dribbling, changing direction 180° | NA,2,7 | COD |  |
|  |  |  |  |  |  |  |  |  |  | regular training | 7weeks | ct |  |
| Eduardo et al., 2015 | 13；13 | 15.33±0.34 | NR | soccer | in-season | 6.2±1.8 | jumps | 60-200,2,9 | PT | regular training | 9weeks | ct | CMJ |
| Senda et al., 2021 | 12；10 | 10.01±0.57 | female | swimmers | competitive | 2.0±1.4 | jumps | 50-120,2,8 | PT | regular training | 8weeks | ct | CMJ, SLJ |
| Mario et al., 2022 | 8；6 | 16.0±2.3 | female | soccer | in-season | 8.8±2.2 | jumps | 64-96,2,4 | PT | regular training | 4weeks | ct | CMJ |
| Eduardo et al., 2008 | 15；10 | 14.7±0.5 | male | basketball | in-season | 5.6±2.6 | leg extension, leg curl, jumps | NR,2,10 | ST+PT | regular training | 10weeks | ct | SJ, CMJ |
| Robert et al., 2003 | 8；9 | 25±4 | male | long distance runners | NR | 10±6 | jumps | 60-180,2-3,6 | PT | regular training | 6weeks | ct | CMJ, 5BT |
| Firas et al., 2019 | 23；8 | 14.5±0.5 | male | soccer | competitive | 5.71±0.40 | leg extension,leg press, jumps | 48-96,2,7 | ST+PT | regular training | 7weeks | ct | SJ, CMJ |
|  |  |  |  |  |  |  | jumps |  | PT |  |  |  |  |
| Hamza et al., 2023 | 12；12 | 14.2±0.3 | male | soccer | competitive | 2< | jumps, dribbling | 72-144,2,8 | PT+COD | regular training | 8weeks | ct | CMJ |
|  | 12；12 | 16.3±0.2 | male | soccer | competitive | 2< | jumps, dribbling |  | PT+COD | regular training |  | ct |  |
| Marco et al., 2018 | 11；8 | 17±0.8 | NR | soccer | NR | NR | drop jumps, short shuttle runs, sprints | 60,2-3,6 | PT+COD | short shuttle runs, sprints | NA,2-3,6 | COD | SLJ |
| Nicolas et al., 2010 | 11；17 | 31±7 | male | long distance runners | NR | NR | drop jump | NR,1,8 | DJ | concentric semisquats | NA,1,8 | ST | CMJ |
|  |  |  |  |  |  |  |  |  |  | regular training | 8weeks | ct |  |
| Ichrak et al., 2020 | 16；10 | 16.4±0.5 | female | basketball | in-season | NR | swiss-ball kneeling hold balancing, single-leg balance, jumps | 80-120,2,8 | BA+PT | regular training | 8weeks | ct | SJ, CMJ |
| Attene et al., 2014 | 18；18 | 14.83±0.92 | female | basketball | NR | NR | jumps | NR,2,6 | PT | regular training | 6weeks | ct | SJ, CMJ |
| Kevin et al., 2009 | 9；8 | 20.2±1.3 | mix | basketball, soccer, volleyball | not in the competitive | NR | back squat, hamstring curl, jumps | NR,3,6 | ST+PT | back squat, hamstring curl | NA,3,6 | ST | CMJ |
| Saharuddin et al., 2018 | 15；30 | 21.33 | NR | soccer | NR | NR | jumps | NR,6,6 | PT | NR | NA,6,6 | ST | CMJ |
|  |  |  |  |  |  |  |  |  |  | regular training | 6weeks | ct |  |
| Sebastian et al., 2018 | 13；6 | 11.0±1.7 | male | basketball | in-season | 1< | drill randomization | NR,2,7 | PT | regular training | 7weeks | ct | CMJ |
|  |  |  |  |  |  |  | jumps |  | PT |  |  |  |  |
| Amanda et al., 2003 | 10；8 | 31±9 | mix | long distance runners | NR | NR | jumps | NR,3,6 | PT | regular training | 6weeks | ct | SJ, CMJ |
| Mehrez et al., 2020 | 26；12 | 16.3±0.4 | male | soccer | in-season | 8< | jumps with extra load | 48-144,2,10 | PT | regular training | 10weeks | ct | SJ, CMJ, 5BT |
|  |  |  |  |  |  |  | jumps |  | PT |  |  |  |  |
| Helmi et al, 2021 | 11;12 | 16.8±0.3 | female | handball | NR | 8.0±1.2 | double-leg stance on a balance board, back half squat, CMJ | NR, 2, 8 | BA+ST+PT | back half squat, CMJ | NR, 2, 8 | ST+PT | CMJ, SJ |
| Chtara et al., 2017 | 10；10 | 13.6±0.3 | male | soccer | competitive season | 5.9±1.2 | jumps | 80-132,2,6 | PT | regular training | 6weeks | ct | SLJ, 5BT |
| Mohamed et al., 2015 | 14；13 | 11.7±1.0 | male | track | in-season | 3.4± 0.6 | jumps | NR,3,10 | PT | regular training | 10weeks | ct | SJ, CMJ, 5BT |
| Carlos et al., 2020 | 4；5 | 20.0±1.08 | male | volleyball | in-season | 5< | jumps with extra load | NR,2,6 | PT | regular training | 6weeks | ct | CMJ |
| Torstein et al., 2016 | 9；7 | 17.2±1.0 | male | ice hockey | NR | NR | lunges, squat, jumps | NR,3,8 | ST+PT | lunges, squat | NA,3,8 | ST | SLJ |
| Cristina et al., 2023 | 23；21 | 10.52±1.90 | female | rhythmic gymnasts | competitive season | NR | passé balance, arabesque balance, jumps | NR,3,8 | BA+PT | regular training | 8weeks | ct | CMJ |
| Hallvard et al., 2022 | 10；11 | 17.1±2.4 | female | handball | NR | NR | jumps | NR,1-2,8 | PT | squats | NA,1-2,8 | ST | CMJ, SLJ |
| Vasileios et al., 2020 | 46；22 | 9.9±1.8 | male | soccer | competitive season | 4.3±2.0 | single leg jumps | 60-120,2,10 | PT | regular training | 10weeks | ct | CMJ, SLJ |
|  |  |  |  |  |  |  | both leg jumps |  | PT |  |  |  |  |
| Mohamed et al., 2014 | 12；11 | 17.1±0.3 | male | handball | in-season | 7.2±1.1 | jumps | NR,2,8 | PT | regular training | 8weeks | ct | SJ, CMJ |
| Francesco et al., 2018 | 10；12 | 13.7±0.5 | male | NR | end of the competitive season | NR | jumps | NR,2,8 | PT | regular training | 8weeks | ct | SJ |
| Felipe et al., 2014 | 17；13 | 15.47±1.28 | male | soccer | in-season | NR | half squat, jumps | NR,2,12 | ST+PT | regular training | 12weeks | ct | CMJ |
| Emma et al., 2016 | 10；10 | 12.5±1.67 | female | competitive gymnasts | NR | 3< | jumps | 54-138,2,6 | PT | regular training | 6weeks | ct | CMJ |
| Hammami et al., 2021 | 17；15 | 16.6±0.5 | male | handball | in-season | 5< | sprint, jumps | NR,NR,8 | HI+PT | regular training | 8weeks | ct | SJ, CMJ |
| Mehrez et al., 2020 | 21；10 | 16.2±0.6 | male | handball | in-season | NR | jumps on sand | 54-108,3,7 | PT | regular training | 7weeks | ct | SJ, CMJ, 5BT |
|  |  |  |  |  |  |  | jumps |  | PT |  |  |  |  |
| Mehrez et al., 2019 | 14；14 | 14.5±0.3 | male | handball | in-season | 4< | jumps, sprint | 48-144,2,8 | PT+COD | regular training | 8weeks | ct | SJ, CMJ, 5BT |
| Kemal et al., 2018 | 13；17 | 16.6±0.6 | female | volleyball | NR | 5< | jumps | NR,2,12 | PT | regular training | 12weeks | ct | CMJ |
| Mohamed et al., 2019 | 24；24 | 11.8±0.4 | male | soccer | in-season | 3.6±0.5 | jumps | 54-124,2,8 | PT | regular training | 8weeks | ct | SJ, CMJ |
| Mohamed et al., 2020 | 14；13 | 19.0±0.9 | male | soccer | in-season | NR | jumps | 140-216,2,6 | PT | regular training | 6weeks | ct | SJ, CMJ |
| Pedro et al., 2017 | 30；28 | 8.72±0.97 | mix | basketball | in-season | 2< | half squat, jumps | 50-130,2,10 | ST+PT | regular training | 10weeks | ct | SJ, CMJ, SLJ |
| Danny et al., 2021 | 9；9 | 32.0±7.3 | mix | endurance runners | NR | NR | jumps | NR,2,6 | PT | isometric mid-thigh pull, isometric ankle plantar flexion | NA,2,6 | ST | CMJ |
| Issam et al., 2018 | 41；16 | 11.29±0.85 | male | soccer | NR | 3< | jumps, oblique shuttle runs | NR,2,8 | PT+COD | regular training | 8weeks | ct | CMJ |
|  |  |  |  |  |  |  | standing with knee on swiss ball, supine straight leg bridge on swiss ball, jumps |  | BA+PT |  |  |  |  |
| Cindy et al., 2013 | 19；16 | 18.94±0.42 | NR | rugby | preseason | 11.26±0.99 | jumps | NR,3,4 | PT | regular training | 4weeks | ct | CMJ |
| Rodrigo et al., 2018 | 49；24 | 13.9±1.9 | male | soccer | in-season | 2< | drop jumps from a box height of 30 cm | 48-90,2,7 | DJ | regular training | 7weeks | ct | CMJ, 5BT |
|  |  |  |  |  |  |  | drop jumps from an individualized box height |  | DJ |  |  |  |  |
| Andrea et al., 2020 | 8；8 | 23±4 | female | soccer | competitive season | NR | jumps | NR,1,12 | PT | regular training | 12weeks | ct | SJ, CMJ, SLJ |
| Ignacio et al., 2018 | 15；7 | 14.6±1.1 | male | basketball | in-season | 4< | jumps | NR,2,6 | PT | regular training | 6weeks | ct | CMJ, SLJ |
|  |  |  |  |  |  |  | jumps |  | PT |  |  |  |  |
| Meszler et al., 2019 | 9；9 | 15.8±1.2 | female | basketball | in-season | 5< | jumps | 61-100,2,7 | PT | regular training | 7weeks | ct | CMJ |
| Gregory et al., 2005 | 10；9 | 15±1 | female | volleyball | preseason | 2< | jumps in water | NR,2,6 | PT | regular training | 6weeks | ct | CMJ |
| Marcin et al., 2021 | 7；8 | 21±3 | female | soccer | NR | 9.75±3.75 | jumps | 50-95,2,4 | PT | regular training | 4weeks | ct | SJ, CMJ |
| Danny et al., 2021 | 11；11 | 37±6 | mix | endurance runners | NR | NR | jumps | NR,2,6 | PT | isometric mid-thigh pull, isometric ankle plantar flexion | NA,2,6 | ST | CMJ |
| Seifeddine et al., 2023 | 28；14 | 25.1±2.3 | male | basketball | competitive season | 12.5±2.9 | jumps, sprint | NR,2,8 | PT+COD | regular training | 8weeks | ct | SJ, CMJ, 5BT |
|  |  |  |  |  |  |  | jumps |  | PT |  |  |  |  |
| Yassine et al., 2016 | 11；23 | 12.8±0.3 | male | soccer | NR | 4.0±1.3 | jumps | 56-140,2,12 | PT | half-squats | NA,2,12 | ST | SJ, CMJ, SLJ, 5BT |
|  |  |  |  |  |  |  |  |  |  | regular training | 12weeks | ct |  |
| Vinícius et al., 2016 | 10；10 | 19.4±1.3 | female | futsal | NR | 3< | jumps | NR,2,4 | PT | regular training | 4weeks | ct | SLJ |
| Nurper et al., 2014 | 9；9 | 18.3±2.6 | female | soccer | NR | 4.2±0.9 | jumps | 90-220,1,8 | PT | regular training | 8weeks | ct | CMJ, SLJ |
| Manibhadra et al., 2022 | 30；30 | 19.06±1.33 | male | badminton | NR | 8.06±1.94 | jumps | 90-120,2,4 | PT | regular training | 4weeks | ct | SJ, SLJ |
| Rodrigo et al., 2020 | 26；12 | 17.1±0.5 | NR | soccer | in-season | 7.3±1.8 | jumps after regular traing | 48-204,2,7 | PT | regular training | 7weeks | ct | SJ, CMJ, SLJ |
|  |  |  |  |  |  |  | jumps before regular traing |  | PT |  |  |  |  |
| Rodrigo et al., 2014 | 17；15 | 22.1±2.7 | mix | middle and long-distance runners |  | 2< | jumps | 60,2,6 | PT | regular training | 6weeks | ct | CMJ |
| Daniel et al., 2022 | 14；14 | 16.07±1.07 | female | volleyball | NR | 4< | jumps | 36-72,2,7 | PT | regular training | 7weeks | ct | CMJ |
| Riadh et al., 2010 | 18；9 | 24.16±0.19 | male | basketball | in-season | 12.4±3.50 | jumps with extra load | NR,2-3,10 | PT | regular training | 10weeks | ct | SJ, CMJ, 5BT |
|  |  |  |  |  |  |  |  |  | PT |  |  |  |  |
| Guillermo et al., 2021 | 10；7 | 21.11±4.16 | female | soccer | NR |  | nordic hamstring exercise, diver, glider, jumps | NR,3,6 | ST+PT | Nordic hamstring exercise, diver, glider | NA,3,6 | ST | CMJ |
| Rodrigo et al., 2015 | 19；19 | 20.5±2.5 | female | NR | competitive | 10.6±3.0 | jumps | 80-160,2,6 | PT | regular training | 6weeks | ct | CMJ |
|  | 21；21 | 20.8±2.7 | male | NR | competitive | 12.0±2.5 | jumps |  | PT | regular training |  | ct |  |
| Juan et al., 2014 | 6；6 | 22.3±8.1 | male | fencers | competitive | 10.3±2.5 | seated calf extension, barbell squat, jumps | NR,2,12 | ST+PT | regular training | 12weeks | ct | SJ, CMJ |
| David et al., 2017 | 10；20 | 24.5±3.4 | NR | soccer | competitive | 8< | full squat, cmj, sprint | NR,2,6 | ST+PT+COD | Full squat | NA,2,6 | ST | CMJ |
|  |  |  |  |  |  |  |  |  |  | regular training | 6weeks | ct |  |
| David et al., 2015 | 15；15 | 12.8±0.5 | NR | soccer | preseason |  | full squat, cmj, sprint | NR,2,6 | ST+PT+COD | regular training | 6weeks | ct | CMJ |
| Senda et al., 2019 | 14；12 | 10.3±0.4 | male | swimmers | in-season | 2.0 ± 1.6 | jumps | 50-120,2,8 | PT | regular training | 8weeks | ct | CMJ, SLJ |
| Ghaith et al., 2021 | 17；16 | 14.6±0.44 | male | soccer | NR | 4.6 ± 0.9 | jumps, sprint | 72-144,2,8 | PT+COD | regular training | 8weeks | ct | SJ, CMJ, SLJ |
| Anis et al., 2014 | 25；26 | 12.1±0.6 | male | basketball | in-season | 2.5 ± 0.5 | jumps | 60-100,2,9 | PT | regular training | 9weeks | ct | SJ, CMJ, 5BT |
| Athanasios et al., 2018 | 12；10 | 14-15 | male | soccer | in-season | NR | barbell cleans, kettlebell snatch, romanian dead lifts, squat, box jumps, sprint | NR,4,5 | WL+PT+ST | regular training | 5weeks | ct | SJ, CMJ |
| Mathias et al., 2012 | 8；8 | 26.0±7.0 | NR | volleyball | NR | NR | electrical muscle stimulation, jumps | NR,2,5 | EMS+PT | calf raises,power cleans, jumps | NR,2,5 | WL+PT+ST | SJ, CMJ |
| Elisa et al., 2011 | 58；20 | 15.9±1.4 | mix | sprinter athlete | NR | 5.64±2.13 | electrostimulation before jumps | NR,2,8 | EMS+PT | jumps | NR,2,8 | PT | CMJ |
|  |  |  |  |  |  |  | electrostimulation after jumps |  | EMS+PT |  |  |  |  |
|  |  |  |  |  |  |  | electrostimulation jumps |  | EMS+PT |  |  |  |  |
| Kevin et al., 2009 | 5；7 | 17.3±0.4 | male | soccer | in-season | 4< | countermovement jump | 80-120,2,6 | CMJ | drop jump | 80-120,2,6 | DJ | CMJ |
| Hechmi et al., 2004 | 8；14 | 21±2 | male | handball | pre-season | 5 | leg press, jumps | NR,NR,6 | ST+PT | leg press | NA,NR,6weeks | ST | SJ, CMJ |
|  |  |  |  |  |  |  |  |  |  | regular training | 6weeks | ct |  |
| Yiannis et al., 2013 | 24；21 | 10.6±0.5 | male | soccer | in-season | 3.6±0.6 | jumps | 60-120,2,12 | PT | regular training | 12weeks | ct | SJ, CMJ, SLJ, 5BT |
| Matauvlj et al., 2001 | 22,11 | 15-16 | male | basketball | in-season | 5~8 | drop jumps from a box height of 50 cm | NR,3,6 | DJ | regular training | 6weeks | ct | CMJ |
|  |  |  |  |  |  |  | drop jumps from a box height of 100 cm |  | DJ |  |  |  |  |
| Marina et al., 2022 | 20；10 | 19.63±1.02 | male | basketball | NR | 2< | jumps on trampoline | NR,3,6 | PT | regular training | 6weeks | ct | CMJ |
|  |  |  |  |  |  |  | jumps |  | PT |  |  |  |  |
| Rodrigo et al., 2019 | 19；20 | 13.5±1.9 | male | soccer | competitive season | 2< | drop jump | 60,2,7 | DJ | regular training | 7weeks | ct | CMJ, 5BT |
| Rodrigo et al., 2018 | 16；7 | 12.6±1.8 | male | soccer | competitive season | 4.0±1.6 | mixed surfaces | 35-90,2,8 | PT | regular training | 8weeks | ct | CMJ, SLJ |
|  |  |  |  |  |  |  | jumps |  | PT |  |  |  |  |
| Alexis et al., 2021 | 10；10 | 12.39±0.56 | male | soccer | competitive season | 5.52±1.21 | jumps | NR,2,6 | PT | regular training | 6weeks | ct | SJ, CMJ |
| Soufiane et al., 2022 | 30；15 | 16.6±0.4 | male | table tennis | in-season | 3< | jumps | NR,2,8 | PT | hang power snatch, hang power clean | NA,2,8 | WL | SJ, CMJ, SLJ |
|  |  |  |  |  |  |  |  |  |  | regular training | 8weeks | ct |  |
| Mehrez et al., 2019 | 21；20 | 13.5±0.3 | female | handball | in-season | 5 | jumps | 60-80,2,9 | PT | regular training | 9weeks | ct | SJ, CMJ, 5BT |
| Tiago et al., 2020 | 16；16 | 11.5±0.9 | male | soccer | NR | 3.7±1.0 | drop jump | NR,2,7 | DJ | regular training | 7weeks | ct | CMJ, 5BT |
|  | 22；22 | 14.5±1.1 | male | soccer | NR | 5.1±2.0 | drop jump |  | DJ | regular training |  | ct |  |
| Yiannis et al., 2018 | 17；14 | 11.8±0.8 | NR | soccer | in-season | NR | jumps, sprint | 100-200,2,6 | PT+COD | regular training | 6weeks | ct | SJ, CMJ, SLJ, 5BT |
| Kike et al., 2023 | 32；8 | 21.2±2.0 | male | basketball | NR | NR | horizontal jumps | 36-72,2,6 | PT | regular training | 6weeks | ct | SJ, CMJ, SLJ |
|  |  |  |  |  |  |  | vertical jumps |  | PT |  |  |  |  |
|  |  |  |  |  |  |  | vertical and horizontal jumps |  | PT |  |  |  |  |

<: At least; 5BT: 5 bounces test; BA+PT: Balance training combined with plyometric training; BA+ST+PT: Complex training consists of balance, strength and plyometrics; CG: Control group; CMJ: Countermovement jump; COD: Change of direction training; ct: Conventional training(technical and tactical training); DJ: Drop jump; EMS+PT: Electrostimulation combined with plyometric training; HI+PT: High-intensity interval training combined with plyometrics; IG: Intervention group; N: Number of participants; NA: Not applicable; NR: Not reported; PT: Plyometric training; PT+COD: Plyometric training combined with change of direction training; SJ: Squat jump; SLJ: Standing long jump; ST: Strength training(traditional resistance training and body weight training); ST+PT: Strength training combined with plyometrics; ST+PT+COD: Complex training consists of strength, plyometrics and change of direction; WL: Weightlifting training; WL+PT+ST: Complex training consists of weight lifting, strength and plyometrics.

B.2. Details of the training intervention.

| Study | Intervention (IG) | | | | | Intervention (CG) | | | | |
| --- | --- | --- | --- | --- | --- | --- | --- | --- | --- | --- |
|  | Type | Duration/min | Sets, repetitions | Intensity | Intermittent time between sets and sessions | Type | Duration/min | Sets, repetitions | Intensity | Intermittent time between sets and sessions |
| Barbara et al., 2017 | PT | 15-20 |  | NR | NR, NR | ct | NR |  | NR | NR, NR |
| Abbas et al., 2018 | DJ | 30-40 |  | max | 120s, 48-72h | ct | 60-70 |  | NR | NR, NR |
|  | DJ |  |  |  |  | ct |  |  |  |  |
|  | DJ |  |  |  |  | ct |  |  |  |  |
| Bent et al., 2008 | ST+PT | NR | 3-5，18-20 | 4-6RM | 60s, NR | ST | 90-120 |  | NR | NR, NR |
|  |  |  |  |  |  | ct |  |  |  |  |
| Fabián et al., 2017 | PT | NR |  | max | NR, 48h | ct | 120 |  | NR | NR, NR |
| Eduardo et al., 2021 | PT | 20 |  | NR | 60s, 24-96h | ST | 55 |  |  | 5min, 24-96h |
|  |  |  |  |  |  | COD |  |  |  |  |
|  |  |  |  |  |  | ct |  |  |  |  |
| Eduardo et al., 2015 | PT | 25 |  | max | 60s, 48-72h | ct | 40 |  | NR | NR, NR |
| Senda et al., 2021 | PT | 25-30 |  | max | 90s, 72h | ct | 80-90 |  | NR | NR, NR |
| Mario et al., 2022 | PT | NR |  | max | 30-60s, 48h | ct | NR |  | NR | NR, NR |
| Eduardo et al., 2008 | ST+PT | NR | 2~3 | 10-12RM | NR, NR | ct | NR |  | NR | NR, NR |
| Robert et al., 2003 | PT | NR |  | NR | NR, NR | ct | NR |  | NR | NR, NR |
| Firas et al., 2019 | ST+PT | 40 | 2-4，6-8 | 30-60%1RM | 3min, 48-72 | ct | 90 |  | NR | NR, NR |
|  | PT |  |  |  |  |  |  |  |  |  |
| Hamza et al., 2023 | PT+COD | NR |  | max | 90s, 24-96h | ct | NR |  | NR | NR, NR |
|  | PT+COD |  |  |  |  | ct |  |  |  |  |
| Marco et al., 2018 | PT+COD | NR |  | NR | NR, 24-96h | COD | NR |  | NR | NR, NR |
| Nicolas et al., 2010 | DJ | NR |  | NR | NR, NR | ST | NR |  | 70-105%PTS | 40-150s, NR |
|  |  |  |  |  |  | ct |  |  |  |  |
| Ichrak et al., 2020 | BA+PT | 45 |  | max | 90s, 24-96h | ct | NR |  | NR | NR, NR |
| Attene et al., 2014 | PT | 20 |  | max | 1min, NR | ct | NR |  | NR | NR, NR |
| Kevin et al., 2009 | ST+PT | NR | 3，10 | 60-65%1RM | NR, 24h | ST | NR | 3，10 | 60-65%1RM | NR, NR |
| Saharuddin et al., 2018 | PT | NR |  | NR | 3-5min, 24h | ST | NR | 2-5，2-5 | 75-95%1RM | 2-5min, NR |
|  |  |  |  |  |  | ct |  |  |  |  |
| Sebastian et al., 2018 | PT | NR |  | max | 60s, 48h | ct | 120 |  | NR | NR, NR |
|  | PT |  |  |  |  |  |  |  |  |  |
| Amanda et al., 2003 | PT | NR |  | NR | NR, NR | ct | NR |  | NR | NR, NR |
| Mehrez et al., 2020 | PT | NR |  | max | 30s-1min, 24-96h | ct | NR |  | NR | NR, NR |
|  | PT |  |  |  |  |  |  |  |  |  |
| Helmi et al, 2021 | BA+ST+PT | NR | 3,8 | 80%1RM | NR, 24-96h | ST+PT | NR | 3,8 | 80%1RM | 2min, 24-96h |
| Chtara et al., 2017 | PT | 20 |  | NR | 90s, NR | ct | 90 |  | NR | NR, NR |
| Mohamed et al., 2015 | PT | 20 |  | max | NR, 24-48h | ct | NR |  | NR | NR, NR |
| Carlos et al., 2020 | PT | 40-50 |  | NR | NR, 48h | ct | 60 |  | NR | NR, NR |
| Torstein et al., 2016 | ST+PT | NR | 3，10 | NR | NR, NR | ST | NR | 3，10 | NR | NR, NR |
| Cristina et al., 2023 | BA+PT | NR | 2-4，6-10 | NR | NR, NR | ct | NR |  | NR | NR, NR |
| Hallvard et al., 2022 | PT | NR |  | max | 3-5min, 48h | ST | NR |  | NR | NR, NR |
| Vasileios et al., 2020 | PT | NR |  | max | NR, 48h | ct | NR |  | NR | NR, NR |
|  | PT |  |  |  |  |  |  |  |  |  |
| Mohamed et al., 2014 | PT | 30 |  | max | NR, 24-96h | ct | NR |  | NR | NR, NR |
| Francesco et al., 2018 | PT | 15 |  | max | 60-90s, 24-96h | ct | NR |  | NR | NR, NR |
| Felipe et al., 2014 | ST+PT | NR | 4-6，6 | max | NR, NR | ct | NR |  | NR | NR, NR |
| Emma et al., 2016 | PT | 40 |  | NR | 60s, NR | ct | NR |  | NR | NR, NR |
| Hammami et al., 2021 | HI+PT | 25-35 |  | max | 10s，3-5min, NR | ct | NR |  | NR | NR, NR |
| Mehrez et al., 2020 | PT | 25 |  | NR | NR, 24-48h | ct | NR |  | NR | NR, NR |
|  | PT |  |  |  |  |  |  |  |  |  |
| Mehrez et al., 2019 | PT+COD | NR |  | NR | 90s, NR | ct | NR |  | NR | NR, NR |
| Kemal et al., 2018 | PT | 20-30 |  | NR | 2-5min, NR | ct | 70 |  | NR | NR, NR |
| Mohamed et al., 2019 | PT | 20-25 |  | NR | NR, 24-96h | ct | NR |  | NR | NR, NR |
| Mohamed et al., 2020 | PT | 23-33 |  | NR | 60s, 24-96h | ct | 100 |  | NR | NR, NR |
| Pedro et al., 2017 | ST+PT | 10~30 |  | max | 90s, 24-96h | ct | NR |  | NR | NR, NR |
| Danny et al., 2021 | PT | NR |  | max | 3min, NR | ST | NR | 2-3，3-5 | NR | 3min, NR |
| Issam et al., 2018 | PT+COD | NR | 1-3，8-12 | max | NR, NR | ct | NR |  | NR | NR, NR |
|  | BA+PT |  |  |  |  |  |  |  |  |  |
| Cindy et al., 2013 | PT | NR |  | NR | 30s, NR | ct | 120 |  | NR | NR, NR |
| Rodrigo et al., 2018 | DJ | 13 |  | max | 30s, 24-96h | ct | 120 |  | NR | NR, NR |
|  | DJ |  |  |  |  |  |  |  |  |  |
| Andrea et al., 2020 | PT | NR |  | NR | NR, NR | ct | NR |  | NR | NR, NR |
| Ignacio et al., 2018 | PT | NR |  | max | 60s, 48h | ct | NR |  | NR | NR, NR |
|  | PT |  |  |  |  |  |  |  |  |  |
| Meszler et al., 2019 | PT | NR |  | max | 2min, 24-96h | ct | NR |  | NR | NR, NR |
| Gregory et al., 2005 | PT | 45 |  | max | NR, NR | ct | NR |  | NR | NR, NR |
| Marcin et al., 2021 | PT | NR |  | max | NR, 48-72h | ct | NR |  | NR | NR, NR |
| Danny et al., 2021 | PT | NR |  | max | 3min, NR | ST | NR |  | NR | NR, NR |
| Seifeddine et al., 2023 | PT+COD | NR |  | max | 3-4min, 24-96h | ct | NR |  | NR | NR, NR |
|  | PT |  |  |  |  |  |  |  |  |  |
| Yassine et al., 2016 | PT | 35-40 |  | max | 90s, 72h | ST | 35-40 | 4，10-12 | 40-60%1RM | 2min, 72h |
|  |  |  |  |  |  | ct |  |  |  |  |
| Vinícius et al., 2016 | PT | 25 |  | NR | NR, NR | ct | NR |  | NR | NR, NR |
| Nurper et al., 2014 | PT | 30-40 |  | low-high | 3-5min, 24h | ct | 120 |  | NR | NR, NR |
| Manibhadra et al., 2022 | PT | 20 |  | NR | NR, 48h | ct | NR |  | NR | NR, NR |
| Rodrigo et al., 2020 | PT | 20 |  | max | 30-60s, 72-96h | ct | 120 |  | NR | NR, NR |
|  | PT |  |  |  |  |  |  |  |  |  |
| Rodrigo et al., 2014 | PT | 30 |  | max | 2min, 48h | ct | NR |  | NR | NR, NR |
| Daniel et al., 2022 | PT | 30 |  | low-moderate | 1-2min, NR | ct | NR |  | NR | NR, NR |
| Riadh et al., 2010 | PT | NR |  | NR | 2-3min, NR | ct | 90 |  | NR | NR, NR |
|  | PT |  |  |  |  |  |  |  |  |  |
| Guillermo et al., 2021 | ST+PT | 20 | 2-3，5-10 | NR | 20-30s, NR | ST | 12 | 2-3，5-10 | 20-30s | 2min, NR |
| Rodrigo et al., 2015 | PT | NR |  | max | 60s, 72h | ct | 120 |  | NR | NR, NR |
|  | PT |  |  |  |  | ct |  |  |  |  |
| Juan et al., 2014 | ST+PT | NR | 3，3-6 | 70-75%1RM | 3min, 24-96h | ct | 60 |  | NR | NR, NR |
| David et al., 2017 | ST+PT+COD | 35 | 2-3，4-6 | 45-58%1RM | 3min, NR | ST | NR |  | NR | NR, NR |
|  |  |  |  |  |  | ct |  |  |  |  |
| David et al., 2015 | ST+PT+COD | 35 | 2-3，4-6 | 45-58%1RM | 3min, 48-72h | ct | NR |  | NR | NR, NR |
| Senda et al., 2019 | PT | 25-30 |  | max | 90s, 72h | ct | 80-90 |  | NR | NR, NR |
| Ghaith et al., 2021 | PT+COD | NR |  | NR | 90s, 24-96h | ct | 90 |  | NR | NR, 24-96h |
| Anis et al., 2014 | PT | 15-25 |  | max | 3min, 24-96h | ct | 90 |  | NR | NR, NR |
| Athanasios et al., 2018 | WL+PT+ST | NR |  | 60-90%1RM | NR, 48-72h | ct | NR |  | NR | NR, NR |
| Mathias et al., 2012 | EMS+PT | 10 | 3，5 | 85%1RM | NR, NR | WL+PT+ST | NR | 3，5 | 85%1RM | NR, NR |
| Elisa et al., 2011 | EMS+PT | NR | 6，8 | NR | NR, NR | PT | NR |  | NR | NR, NR |
|  | EMS+PT |  |  |  |  |  |  |  |  |  |
|  | EMS+PT |  |  |  |  |  |  |  |  |  |
| Kevin et al., 2009 | CMJ | NR |  | max | NR, NR | DJ | NR |  | NR | NR, NR |
| Hechmi et al., 2004 | ST+PT | NR | 6，10 | 70%1RM | 3min, NR | ST | NR | 6，10 | 70%1RM | 3min, NR |
|  |  |  |  |  |  | ct |  |  |  |  |
| Yiannis et al., 2013 | PT | 20-25 |  | max | 90-180s, 72h | ct | NR |  | NR | NR, NR |
| Matauvlj et al., 2001 | DJ | 15 |  | NR | 3min, NR | ct | 90 |  | NR | NR, NR |
|  | DJ |  |  |  |  |  |  |  |  |  |
| Marina et al., 2022 | PT | 15 |  | NR | 10-60s, NR | ct | NR |  | NR | NR, NR |
|  | PT |  |  |  |  |  |  |  |  |  |
| Rodrigo et al., 2019 | DJ | 20 |  | max | 90s, 48h | ct | 90 |  | NR | NR, NR |
| Rodrigo et al., 2018 | PT | 10~15 |  | max | 30-60s, 72-96h | ct | 90 |  | NR | NR, NR |
|  | PT |  |  |  |  |  |  |  |  |  |
| Alexis et al., 2021 | PT | 20-35 |  | max | 120s, 48h | ct | 80-90 |  | NR | NR, NR |
| Soufiane et al., 2022 | PT | NR |  | max | 3-5min, 24-96h | WL | NR |  | NR | NR, NR |
|  |  |  |  |  |  | ct |  |  |  |  |
| Mehrez et al., 2019 | PT | NR |  | max | 90s, 24-96h | ct | NR |  | NR | NR, NR |
| Tiago et al., 2020 | DJ | 21 |  | max | 90s, 24-96h | ct | 90 |  | NR | NR, NR |
|  | DJ |  |  |  |  | ct |  |  |  |  |
| Yiannis et al., 2018 | PT+COD | 15-20 |  | max | NR, 72h | ct | 90 |  | NR | NR, NR |
| Kike et al., 2023 | PT | NR |  | NR | 3min, NR | ct | NR |  | NR | NR, NR |
|  | PT |  |  |  |  |  |  |  |  |  |
|  | PT |  |  |  |  |  |  |  |  |  |

Note: PTS: Peak treadmill speed.

**Supplementary Appendix C.** League table

C.1. League table on SJ.

| HI+PT | WL | WL+PT+ST | ST | ST+PT | PT+COD | BA+PT | PT | EMS+PT | ct |
| --- | --- | --- | --- | --- | --- | --- | --- | --- | --- |
| HI+PT | -1.05 (-2.82,0.71) | -1.29 (-3.24,0.66) | -1.33 (-2.88,0.22) | -1.46 (-2.95,0.03) | -1.49 (-3.01,0.02) | **-1.94 (-3.82,-0.06)** | **-2.01 (-3.43,-0.60)** | **-2.51 (-4.95,-0.07)** | **-2.75 (-4.14,-1.36)** |
| 1.05 (-0.71,2.82) | WL | -0.24 (-1.98,1.51) | -0.28 (-1.56,1.00) | -0.41 (-1.62,0.80) | -0.44 (-1.68,0.80) | -0.89 (-2.56,0.78) | -0.96 (-2.05,0.12) | -1.46 (-3.74,0.82) | **-1.70 (-2.78,-0.61)** |
| 1.29 (-0.66,3.24) | 0.24 (-1.51,1.98) | WL+PT+ST | -0.05 (-1.57,1.48) | -0.17 (-1.64,1.30) | -0.21 (-1.70,1.29) | -0.65 (-2.52,1.21) | -0.73 (-2.11,0.66) | -1.22 (-2.69,0.24) | **-1.46 (-2.83,-0.10)** |
| 1.33 (-0.22,2.88) | 0.28 (-1.00,1.56) | 0.05 (-1.48,1.57) | ST | -0.13 (-0.88,0.62) | -0.16 (-1.07,0.75) | -0.61 (-2.05,0.83) | -0.68 (-1.38,0.02) | -1.18 (-3.29,0.94) | **-1.42 (-2.10,-0.73)** |
| 1.46 (-0.03,2.95) | 0.41 (-0.80,1.62) | 0.17 (-1.30,1.64) | 0.13 (-0.62,0.88) | ST+PT | -0.03 (-0.84,0.77) | -0.48 (-1.86,0.90) | -0.55 (-1.13,0.02) | -1.05 (-3.13,1.02) | **-1.29 (-1.83,-0.74)** |
| 1.49 (-0.02,3.01) | 0.44 (-0.80,1.68) | 0.21 (-1.29,1.70) | 0.16 (-0.75,1.07) | 0.03 (-0.77,0.84) | PT+COD | -0.45 (-1.85,0.96) | -0.52 (-1.15,0.12) | -1.02 (-3.11,1.07) | **-1.25 (-1.86,-0.65)** |
| 1.94 (0.06,3.82) | 0.89 (-0.78,2.56) | 0.65 (-1.21,2.52) | 0.61 (-0.83,2.05) | 0.48 (-0.90,1.86) | 0.45 (-0.96,1.85) | BA+PT | -0.07 (-1.36,1.22) | -0.57 (-2.94,1.80) | -0.81 (-2.07,0.46) |
| 2.01 (0.60,3.43) | 0.96 (-0.12,2.05) | 0.73 (-0.66,2.11) | 0.68 (-0.02,1.38) | 0.55 (-0.02,1.13) | 0.52 (-0.12,1.15) | 0.07 (-1.22,1.36) | PT | -0.50 (-2.51,1.52) | -0.73 (-0.97,-0.50) |
| 2.51 (0.07,4.95) | 1.46 (-0.82,3.74) | 1.22 (-0.24,2.69) | 1.18 (-0.94,3.29) | 1.05 (-1.02,3.13) | 1.02 (-1.07,3.11) | 0.57 (-1.80,2.94) | 0.50 (-1.52,2.51) | EMS+PT | -0.24 (-2.24,1.76) |
| 2.75 (1.36,4.14) | 1.70 (0.61,2.78) | 1.46 (0.10,2.83) | 1.42 (0.73,2.10) | 1.29 (0.74,1.83) | 1.25 (0.65,1.86) | 0.81 (-0.46,2.07) | 0.73 (0.50,0.97) | 0.24 (-1.76,2.24) | ct |

C.2. League table on CMJ.

| EMS+PT | HI+PT | WL+PT+ST | WL | BA+PT | PT+COD | ST+PT | PT | ST+PT+COD | BA+ST+PT | DJ | CMJ | ST | COD | ct |
| --- | --- | --- | --- | --- | --- | --- | --- | --- | --- | --- | --- | --- | --- | --- |
| EMS+PT | -0.59 (-2.39,1.22) | -1.04 (-2.22,0.14) | -1.50 (-3.03,0.04) | **-2.32 (-3.52,-1.13)** | **-2.57 (-3.63,-1.51)** | **-2.67 (-3.72,-1.62)** | **-2.70 (-3.62,-1.79)** | **-2.81 (-4.12,-1.49)** | **-2.95 (-4.72,-1.19)** | **-3.00 (-4.04,-1.97)** | **-3.29 (-5.22,-1.35)** | **-3.08 (-4.08,-2.08)** | **-3.21 (-4.72,-1.71)** | **-3.45 (-4.38,-2.52)** |
| 0.59 (-1.22,2.39) | HI+PT | -0.45 (-2.40,1.50) | -0.91 (-2.88,1.07) | **-1.74 (-3.45,-0.02)** | **-1.98 (-3.61,-0.36)** | **-2.08 (-3.71,-0.45)** | **-2.11 (-3.67,-0.56)** | **-2.22 (-4.03,-0.41)** | **-2.37 (-4.52,-0.21)** | **-2.42 (-4.03,-0.80)** | **-2.70 (-4.99,-0.40)** | **-2.49 (-4.09,-0.90)** | **-2.63 (-4.58,-0.67)** | **-2.86 (-4.41,-1.32)** |
| 1.04 (-0.14,2.22) | 0.45 (-1.50,2.40) | WL+PT+ST | -0.46 (-2.16,1.24) | -1.29 (-2.69,0.12) | **-1.54 (-2.83,-0.25)** | **-1.63 (-2.92,-0.35)** | **-1.67 (-2.85,-0.48)** | **-1.77 (-3.28,-0.26)** | **-1.92 (-3.83,-0.00)** | **-1.97 (-3.24,-0.69)** | **-2.25 (-4.32,-0.18)** | **-2.04 (-3.29,-0.80)** | **-2.18 (-3.86,-0.50)** | **-2.41 (-3.60,-1.23)** |
| 1.50 (-0.04,3.03) | 0.91 (-1.07,2.88) | 0.46 (-1.24,2.16) | WL | -0.83 (-2.27,0.61) | -1.08 (-2.41,0.25) | -1.17 (-2.50,0.15) | -1.21 (-2.43,0.02) | -1.31 (-2.85,0.23) | -1.46 (-3.40,0.48) | **-1.51 (-2.82,-0.19)** | -1.79 (-3.89,0.30) | **-1.59 (-2.87,-0.30)** | **-1.72 (-3.43,-0.01)** | **-1.95 (-3.18,-0.72)** |
| 2.32 (1.13,3.52) | 1.74 (0.02,3.45) | 1.29 (-0.12,2.69) | 0.83 (-0.61,2.27) | BA+PT | -0.25 (-1.09,0.59) | -0.35 (-1.25,0.56) | -0.38 (-1.15,0.39) | -0.48 (-1.68,0.71) | -0.63 (-2.31,1.05) | -0.68 (-1.56,0.20) | -0.96 (-2.82,0.89) | -0.76 (-1.61,0.09) | -0.89 (-2.30,0.52) | **-1.13 (-1.87,-0.38)** |
| 2.57 (1.51,3.63) | 1.98 (0.36,3.61) | 1.54 (0.25,2.83) | 1.08 (-0.25,2.41) | 0.25 (-0.59,1.09) | PT+COD | -0.10 (-0.82,0.62) | -0.13 (-0.67,0.41) | -0.24 (-1.30,0.83) | -0.38 (-1.97,1.21) | -0.43 (-1.12,0.26) | -0.71 (-2.49,1.06) | -0.51 (-1.16,0.14) | -0.64 (-1.94,0.65) | **-0.88 (-1.39,-0.37)** |
| 2.67 (1.62,3.72) | 2.08 (0.45,3.71) | 1.63 (0.35,2.92) | 1.17 (-0.15,2.50) | 0.35 (-0.56,1.25) | 0.10 (-0.62,0.82) | ST+PT | -0.03 (-0.56,0.50) | -0.14 (-1.19,0.91) | -0.29 (-1.70,1.13) | -0.33 (-1.01,0.34) | -0.62 (-2.39,1.15) | -0.41 (-0.97,0.14) | -0.55 (-1.83,0.74) | **-0.78 (-1.29,-0.27)** |
| 2.70 (1.79,3.62) | 2.11 (0.56,3.67) | 1.67 (0.48,2.85) | 1.21 (-0.02,2.43) | 0.38 (-0.39,1.15) | 0.13 (-0.41,0.67) | 0.03 (-0.50,0.56) | PT | -0.11 (-1.06,0.85) | -0.25 (-1.76,1.26) | -0.30 (-0.80,0.20) | -0.58 (-2.29,1.12) | -0.38 (-0.80,0.04) | -0.51 (-1.71,0.68) | **-0.75 (-0.94,-0.55)** |
| 2.81 (1.49,4.12) | 2.22 (0.41,4.03) | 1.77 (0.26,3.28) | 1.31 (-0.23,2.85) | 0.48 (-0.71,1.68) | 0.24 (-0.83,1.30) | 0.14 (-0.91,1.19) | 0.11 (-0.85,1.06) | ST+PT+COD | -0.15 (-1.91,1.62) | -0.20 (-1.23,0.84) | -0.48 (-2.41,1.46) | -0.27 (-1.25,0.70) | -0.41 (-1.91,1.10) | -0.64 (-1.58,0.29) |
| 2.95 (1.19,4.72) | 2.37 (0.21,4.52) | 1.92 (0.00,3.83) | 1.46 (-0.48,3.40) | 0.63 (-1.05,2.31) | 0.38 (-1.21,1.97) | 0.29 (-1.13,1.70) | 0.25 (-1.26,1.76) | 0.15 (-1.62,1.91) | BA+ST+PT | -0.05 (-1.62,1.52) | -0.33 (-2.60,1.93) | -0.13 (-1.65,1.40) | -0.26 (-2.17,1.65) | -0.49 (-2.00,1.01) |
| 3.00 (1.97,4.04) | 2.42 (0.80,4.03) | 1.97 (0.69,3.24) | 1.51 (0.19,2.82) | 0.68 (-0.20,1.56) | 0.43 (-0.26,1.12) | 0.33 (-0.34,1.01) | 0.30 (-0.20,0.80) | 0.20 (-0.84,1.23) | 0.05 (-1.52,1.62) | DJ | -0.28 (-1.92,1.35) | -0.08 (-0.67,0.52) | -0.21 (-1.49,1.06) | -0.45 (-0.91,0.02) |
| 3.29 (1.35,5.22) | 2.70 (0.40,4.99) | 2.25 (0.18,4.32) | 1.79 (-0.30,3.89) | 0.96 (-0.89,2.82) | 0.71 (-1.06,2.49) | 0.62 (-1.15,2.39) | 0.58 (-1.12,2.29) | 0.48 (-1.46,2.41) | 0.33 (-1.93,2.60) | 0.28 (-1.35,1.92) | CMJ | 0.21 (-1.53,1.94) | 0.07 (-2.00,2.14) | -0.16 (-1.86,1.53) |
| 3.08 (2.08,4.08) | 2.49 (0.90,4.09) | 2.04 (0.80,3.29) | 1.59 (0.30,2.87) | 0.76 (-0.09,1.61) | 0.51 (-0.14,1.16) | 0.41 (-0.14,0.97) | 0.38 (-0.04,0.80) | 0.27 (-0.70,1.25) | 0.13 (-1.40,1.65) | 0.08 (-0.52,0.67) | -0.21 (-1.94,1.53) | ST | -0.13 (-1.35,1.08) | -0.37 (-0.78,0.04) |
| 3.21 (1.71,4.72) | 2.63 (0.67,4.58) | 2.18 (0.50,3.86) | 1.72 (0.01,3.43) | 0.89 (-0.52,2.30) | 0.64 (-0.65,1.94) | 0.55 (-0.74,1.83) | 0.51 (-0.68,1.71) | 0.41 (-1.10,1.91) | 0.26 (-1.65,2.17) | 0.21 (-1.06,1.49) | -0.07 (-2.14,2.00) | 0.13 (-1.08,1.35) | COD | -0.23 (-1.43,0.96) |
| 3.45 (2.52,4.38) | 2.86 (1.32,4.41) | 2.41 (1.23,3.60) | 1.95 (0.72,3.18) | 1.13 (0.38,1.87) | 0.88 (0.37,1.39) | 0.78 (0.27,1.29) | 0.75 (0.55,0.94) | 0.64 (-0.29,1.58) | 0.49 (-1.01,2.00) | 0.45 (-0.02,0.91) | 0.16 (-1.53,1.86) | 0.37 (-0.04,0.78) | 0.23 (-0.96,1.43) | ct |

C.3. League table on SLJ.

| WL | PT | PT+COD | ST | BA+ST+PT | ST+PT | COD | ct |
| --- | --- | --- | --- | --- | --- | --- | --- |
| WL | -0.54 (-1.91,0.83) | -0.55 (-2.31,1.21) | -0.78 (-2.43,0.88) | -0.81 (-3.22,1.60) | -0.81 (-2.61,1.00) | -1.09 (-3.51,1.33) | -1.24 (-2.61,0.13) |
| 0.54 (-0.83,1.91) | PT | -0.01 (-1.18,1.16) | -0.24 (-1.20,0.73) | -0.27 (-2.27,1.74) | -0.27 (-1.48,0.95) | -0.55 (-2.58,1.47) | **-0.70 (-1.06,-0.33)** |
| 0.55 (-1.21,2.31) | 0.01 (-1.16,1.18) | PT+COD | -0.23 (-1.71,1.25) | -0.26 (-2.54,2.02) | -0.26 (-1.88,1.37) | -0.54 (-2.20,1.12) | -0.69 (-1.79,0.42) |
| 0.78 (-0.88,2.43) | 0.24 (-0.73,1.20) | 0.23 (-1.25,1.71) | ST | -0.03 (-2.06,2.00) | -0.03 (-1.28,1.22) | -0.32 (-2.54,1.91) | -0.46 (-1.44,0.52) |
| 0.81 (-1.60,3.22) | 0.27 (-1.74,2.27) | 0.26 (-2.02,2.54) | 0.03 (-2.00,2.06) | BA+ST+PT | 0.00 (-1.60,1.60) | -0.29 (-3.11,2.53) | -0.43 (-2.42,1.56) |
| 0.81 (-1.00,2.61) | 0.27 (-0.95,1.48) | 0.26 (-1.37,1.88) | 0.03 (-1.22,1.28) | -0.00 (-1.60,1.60) | ST+PT | -0.29 (-2.61,2.03) | -0.43 (-1.62,0.76) |
| 1.09 (-1.33,3.51) | 0.55 (-1.47,2.58) | 0.54 (-1.12,2.20) | 0.32 (-1.91,2.54) | 0.29 (-2.53,3.11) | 0.29 (-2.03,2.61) | COD | -0.14 (-2.14,1.85) |
| 1.24 (-0.13,2.61) | 0.70 (0.33,1.06) | 0.69 (-0.42,1.79) | 0.46 (-0.52,1.44) | 0.43 (-1.56,2.42) | 0.43 (-0.76,1.62) | 0.14 (-1.85,2.14) | ct |

C.4. League table on 5BT.

| ST | PT | DJ | PT+COD | ct |
| --- | --- | --- | --- | --- |
| ST | -0.40 (-1.73,0.92) | -0.90 (-2.37,0.57) | -1.08 (-2.63,0.46） | **-1.39 (-2.71,-0.06)** |
| 0.40 (-0.92,1.73) | PT | -0.49 (-1.25,0.27) | -0.68 (-1.55,0.20） | **-0.98 (-1.40,-0.56)** |
| 0.90 (-0.57,2.37) | 0.49 (-0.27,1.25) | DJ | -0.18 (-1.22,0.85） | -0.49 (-1.12,0.14) |
| 1.08 (-0.46,2.63) | 0.68 (-0.20,1.55) | 0.18 (-0.85,1.22) | PT+COD | -0.31 (-1.12,0.51) |
| 1.39 (0.06,2.71) | 0.98 (0.56,1.40) | 0.49 (-0.14,1.12) | 0.31 (-0.51,1.12） | ct |

**Supplementary Appendix D.** Sensitivity analysis

D.1. Sensitivity analysis of SJ


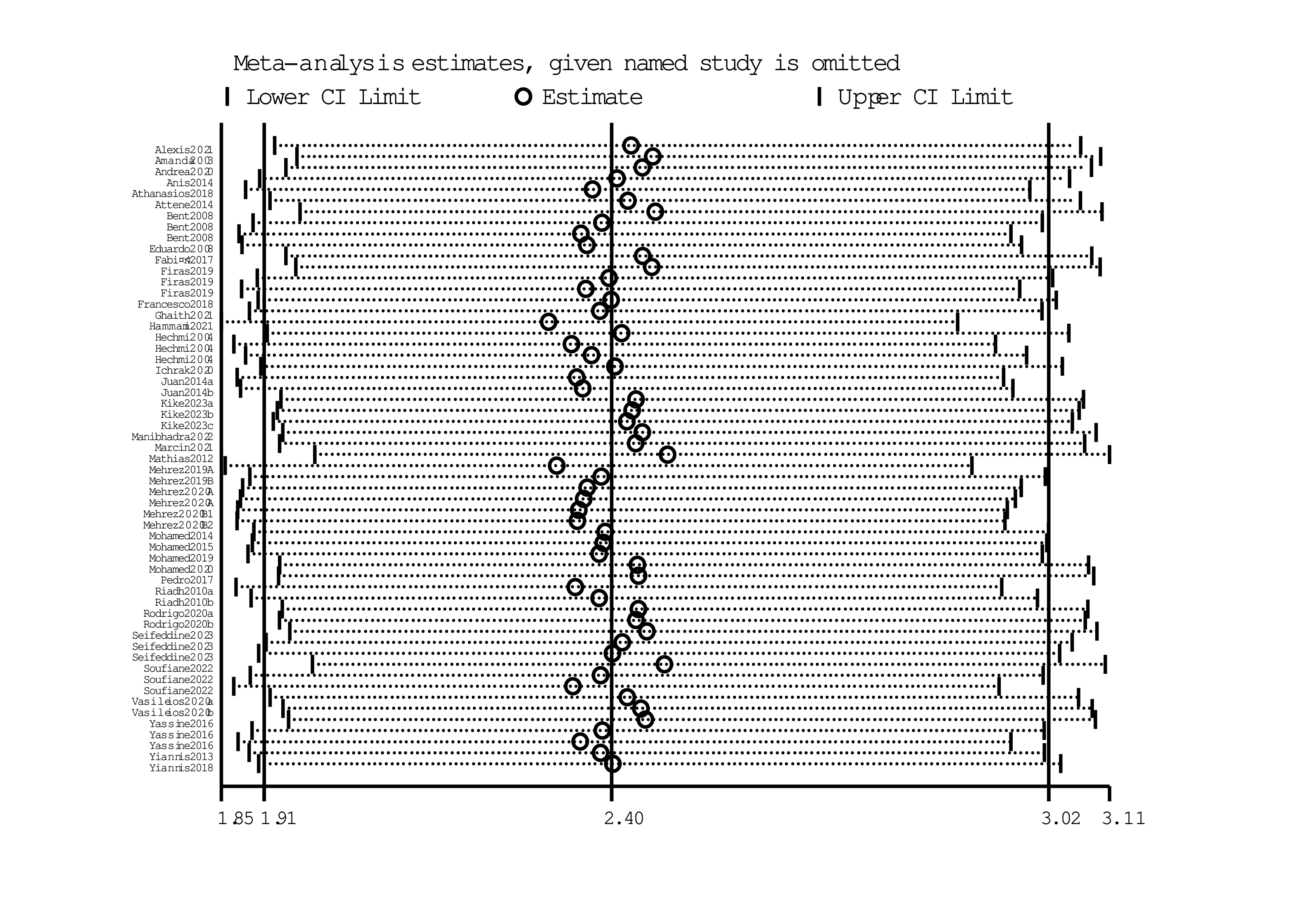


D.2. Sensitivity analysis of CMJ


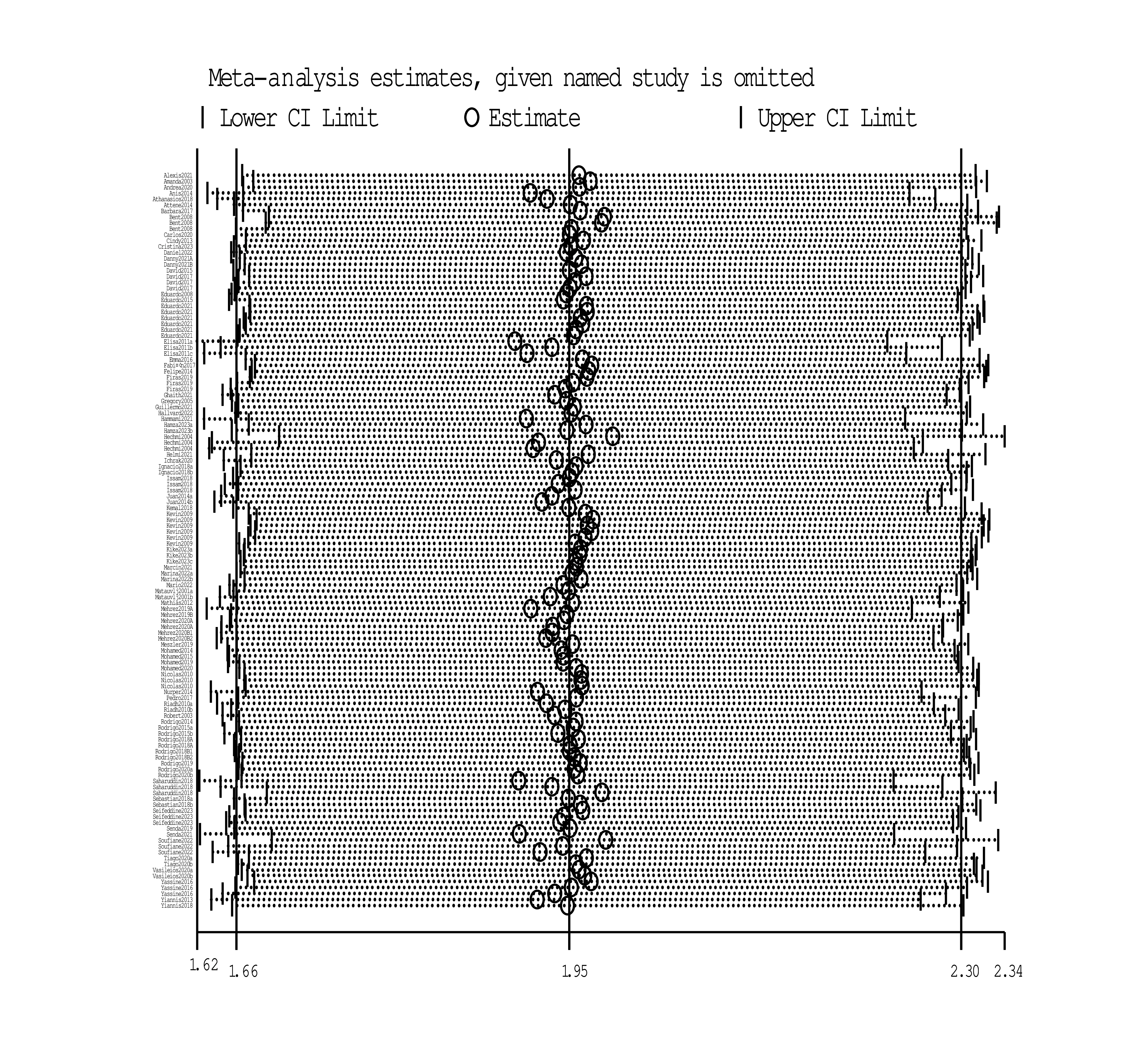


D.3. Sensitivity analysis of SLJ


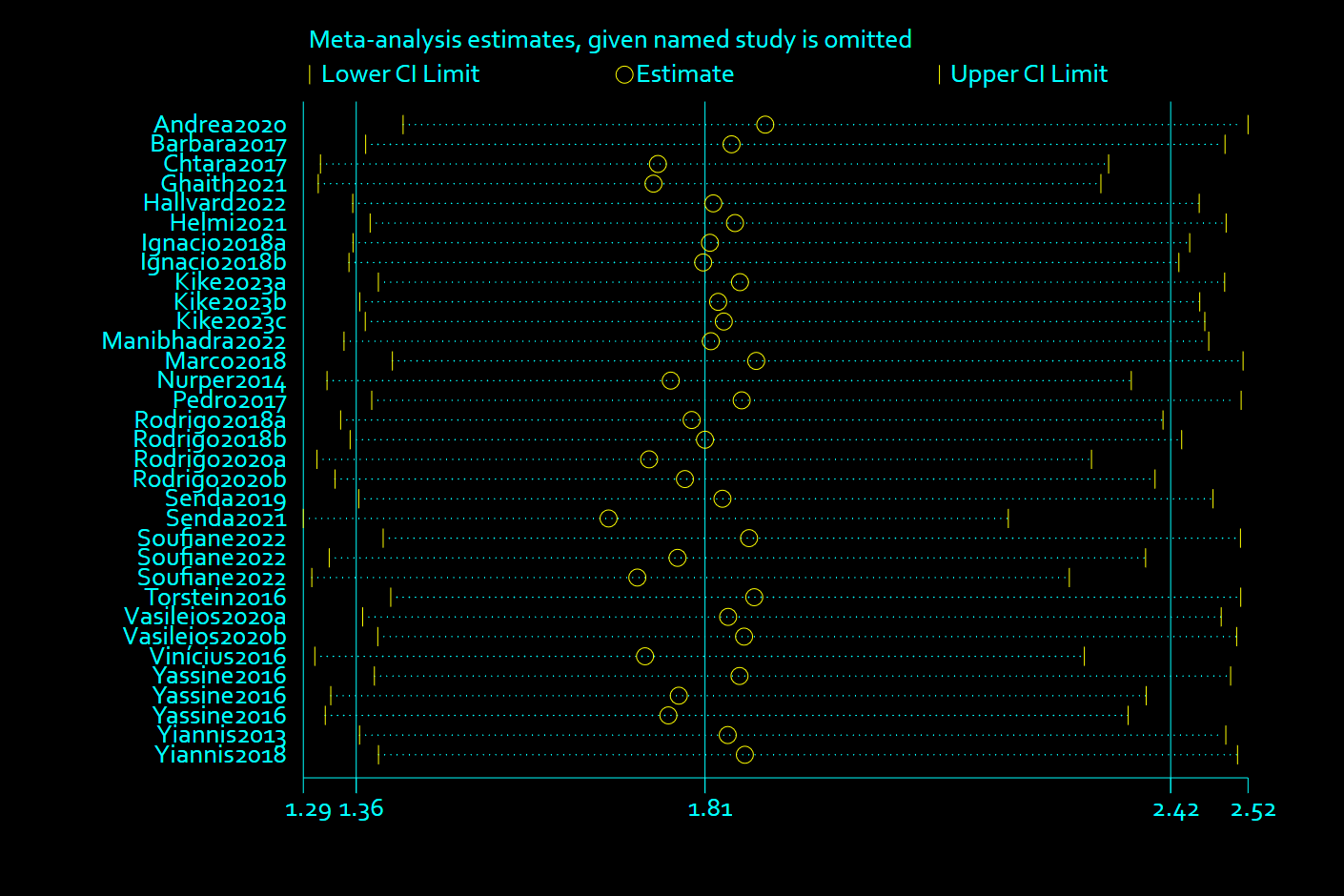


D.4. Sensitivity analysis of 5BT


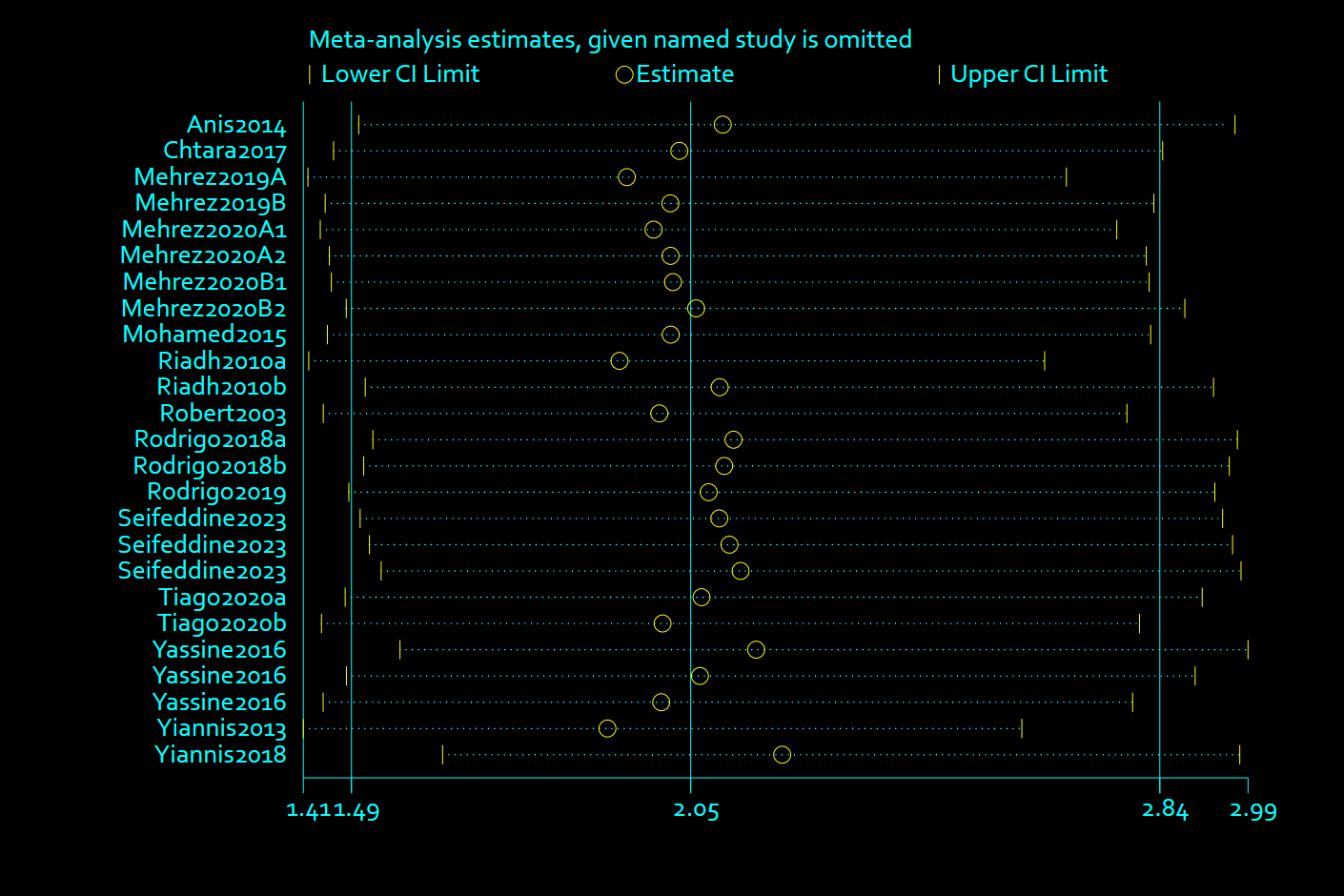

Supplement: Supplementary file 1 [file Table1.DOCX]
